# Inhibition of phospholipase D1 induces immunogenic cell death and potentiates cancer immunotherapy in colorectal cancer

**DOI:** 10.1038/s12276-022-00853-6

**Published:** 2022-09-21

**Authors:** Won Chan Hwang, Doona Song, Hyesung Lee, Changmok Oh, Seong Hun Lim, Hyeon Jeong Bae, Nam Doo Kim, Gyoonhee Han, Do Sik Min

**Affiliations:** 1 Department of Pharmacy, Yonsei University, Incheon, 21983 Republic of Korea; 2 Graduate Program of Industrial Pharmaceutical Science, Yonsei University, Incheon, 21983 Republic of Korea; 3 Department of Biotechnology, College of Life Science and Biotechnology, Yonsei University, Seoul, 03722 Republic of Korea; 4 Voronoi Bio Inc., 32 Songdokwahak-ro, Yeonsu-gu, Incheon, 21984 Republic of Korea; 5 Yonsei Institute of Pharmaceutical Sciences, College of Pharmacy, Yonsei University, Incheon, 21983 Republic of Korea

**Keywords:** Colon cancer, Cancer microenvironment

## Abstract

Phospholipase D (PLD) is a potential therapeutic target against cancer. However, the contribution of PLD inhibition to the antitumor response remains unknown. We developed a potent and selective PLD1 inhibitor based on computer-aided drug design. The inhibitor enhanced apoptosis in colorectal cancer (CRC) cells but not in normal colonic cells, and in vitro cardiotoxicity was not observed. The inhibitor downregulated the Wnt/β-catenin signaling pathway and reduced the migration, invasion, and self-renewal capacity of CRC cells. In cancer, therapeutic engagement of immunogenic cell death (ICD) leads to more effective responses by eliciting the antitumor immunity of T cells. The CRC cells treated with the inhibitor showed hallmarks of ICD, including downregulation of “do not eat-me” signals (CD24, CD47, programmed cell death ligand 1 [PD-L1]), upregulation of “eat-me” signal (calreticulin), release of high-mobility group Box 1, and ATP. PLD1 inhibition subsequently enhanced the phagocytosis of cancer cells by macrophages through the surface expression of costimulatory molecules; as a result, the cancer cells were more susceptible to cytotoxic T-cell-mediated killing. Moreover, PLD1 inhibition attenuated colitis-associated CRC and orthotopically injected tumors, probably by controlling multiple pathways, including Wnt signaling, phagocytosis checkpoints, and immune signaling. Furthermore, combination therapy with a PLD1 inhibitor and an anti-PD-L1 antibody further enhanced tumor regression via immune activation in the tumor environment. Collectively, in this study, PLD1 was identified as a critical regulator of the tumor microenvironment in colorectal cancer, suggesting the potential of PLD1 inhibitors for cancer immunotherapy based on ICD and immune activation. PLD1 inhibitors may act as promising immune modulators in antitumor treatment via ICD.

## Colorectal cancer: Enzyme inhibition enhances immunotherapy

A novel drug that can inhibit an enzyme involved in colorectal cancer progression shows promise in trials on mouse models. The phospholipase D1 (PLD1) enzyme reinforces a critical signaling pathway that promotes cancer progression and drug resistance. Using computer-aided drug design, South Korean researchers led by Do Sik Min and Gyoonhee Han at Yonsei University in Incheon and Seoul, respectively, have developed a drug that specifically binds to and inhibits PLD1. In trials, the researchers observed downregulation of PLD1’s associated signaling pathway, and reductions in the ability of colorectal cancer cells to migrate, invade and replicate. The drug suppressed the cancer cells’ “don’t-eat-me” signals and upregulated “eat-me” signals, triggering improved responses from the immune system. The drug was even more effective when used in combination with an immunotherapy agent.

## Introduction

Phospholipase D (PLD) catalyzes phospholipid hydrolysis to generate phosphatidic acid (PA), a lipid secondary messenger. The predominant human isozymes are PLD1 and PLD2, which are phosphatidylcholine-specific enzymes^1^. PLD has been identified as a therapeutic target for several diseases, including cancer (most prominently), inflammation, neurodegenerative, and cardiovascular diseases, prompting interest in discovering small-molecule inhibitors^2^. Potent small-molecule inhibitors of PLD have been developed^3^, and pharmacological inhibition is effective in preventing cancer cell invasion in cell cultures, neoangiogenesis, and metastasis^2,4^. PLD1 drives a positive feedback loop to reinforce the Wnt/β-catenin signaling axis^5,6^, which is involved in cancer progression, drug resistance, and immune escape^7^. However, it remains unknown whether the immune microenvironment is associated with the function of PLD. Inducing cancer cell apoptosis by inhibiting PLD may enhance the immunogenicity of tumor cells and provide an opportunity for generating tumor immunity. Apoptotic cancer cells can trigger immunogenic cell death (ICD), in which dead or dying cells are taken up by macrophages and processed as tumor-associated antigens to activate adaptive immunity^8^. Phagocytosis checkpoints, which are ‘do not eat-me’ signals caused by anti-phagocytic surface proteins, are implicated in mediating the evasion of phagocytic clearance by tumor cells^9,10^. In many cancers, malignant cells express a “do not eat-me” signal at higher levels than those in normal cells; this potentially occurs to balance the increased expression of pro-phagocytosis ‘eat me’ signals, such as calreticulin (CRT)^9^. Thus, cell-surface expression of CRT, together with secretion of ATP and release of high-mobility group protein B1 (HMGB1), defines the damage-associated molecular patterns (DAMPs) necessary to elicit ICD^11^. Therefore, therapeutically targeting phagocytosis checkpoints in cancer might provide a new avenue for developing cancer immunotherapy to eliminate tumor immune evasion. PLD1 depletion in the tumor microenvironment results in lower tumor burden and reduced metastasis^4^. Targeting PLD1 led to decreased macrophage function and neutrophil migration to tumor sites^12^. PLD1 is also important for signaling mediated by T-cell receptors^13^ and lymphocyte adhesion and migration^14,15^. However, the functional role of PLD1 in the link between PLD1-mediated immune responses and tumor immunity has not been elucidated. Ligand-based drug design has led to the discovery of universal and isozyme-selective small-molecule inhibitors of PLD^3,16^. Recently, the crystal structures of human PLD1 (hPLD1) and hPLD2 were identified, enabling the drug design and characterization of isozyme selectivity^17,18^. In this study, we report a newly developed inhibitor based on the hPLD1 structure and evaluated its selectivity for PLD1 inhibition. We further assessed PLD1 inhibitor-treated tumors for different hallmarks of ICD, phagocytosis and T-cell immune signaling. An effective PLD1 inhibitor with effects on tumor regression and immune activation in the tumor environment might offer a promising new treatment modality against cancer.

## Materials and methods

### Reagents

Purified recombinant Wnt3a (R&D Systems) and 5-fluorouracil (5-FU, Sigma-Aldrich) were commercially obtained. The PLD1 inhibitor (VU0155069) was purchased from Cayman Chemical (Ann Arbor, MI, USA). A dual-luciferase assay kit was purchased from Promega. Azoxymethane (AOM) and dextran sodium sulfate (DSS, 35,000–50,000 Da) were purchased from Sigma-Aldrich and MP Biomedicals, respectively.

### Cell lines and cell culture

Colon cancer cells (DLD1, HCT116, SW480, RKO, and MC38) and HEK293 cells were purchased from ATCC and cultured in DMEM (Corning) or RPMI1640 (Corning) supplemented with 10% FBS at 37 °C in a humidified atmosphere containing 5% CO_2_. To establish an MC38 cell line expressing GFP-Luciferase, the MC38 cells were transduced with the retrovirus LPCX-GFP-Luciferase. All cell lines were used within 15 passages from thawing and were routinely tested for mycoplasma contamination using the Myco-Read Mycoplasma Detection Kit (BIOMAX). The normal colon epithelial cell line (FHC) was obtained from ATCC and cultured in a 1:1 mixture of Ham F-12 medium and DMEM supplemented with 25 mmol/L HEPES, 10 ng/mL cholera toxin (Calbiochem), 0.005 mg/mL insulin, 0.005 mg/mL transferrin, 100 ng/mL hydrocortisone (Sigma-Aldrich), and 10% FBS. PLD^+/+^, PLD1^−/−^, and PLD2^−/−^ mouse embryonic fibroblasts (MEFs) were obtained from 14-day embryos of PLD^+/+^, PLD1^−/−^, and PLD2^−/−^ mice, respectively. Immortalized MEFs were obtained by performing serial passaging with primary MEFs until they surpassed their growth crisis stage.

### Phospholipase D activity assay

Cells were labeled with [H^3^] myristic acid, and PLD activity was assessed by measuring the formation of [H^3^] phosphatidylbutanol, which is the product of PLD-mediated transphosphatidylation, in the presence of 1-butanol, as previously described^19^.

### Streptavidin pull-down assay

Cells were lysed in passive lysis buffer (Promega). Cell lysates were incubated with biotinylated chemicals (A4333, 10 μM) or biotin (10 μM) at 37 °C for 1 h and treated with streptavidin beads (Thermo Scientific) at 20 °C for 1 h. After removing free biotinylated chemicals by washing with PBS, the samples were subjected to SDS‒PAGE followed by immunoblotting with an antibody against PLD^20^.

### Cell viability

Cell viability was measured by MTT (3-(4,5-dimethylthiazol2-yl)-2,5-diphenyltetrazolium bromide, Sigma-Aldrich). Cells (5 × 10^3^ cells/well) were seeded in a 96-well plate. After 24 h, cells were treated with 0.5, 1, 2, 5 or 10 μM compounds for 48 h or 2 μg/mL compounds for 12, 24, 48 or 72 h. After treatment, the cells were incubated with 0.5 mg/mL MTT in the media for 2 h and lysed with DMSO. Absorbance was measured at 570 nm using a VersaMax ELISA microplate reader (Molecular Devices).

### Cell cycle and apoptosis assay

For cell cycle analysis, the cells were treated with Α3373 or 5-FU. The cells were labeled with 10 μΜ bromodeoxyuridine (BrdU) for 2 h. Subsequently, the cells were stained with 7-aminoactinomycin D (7-AAD, Invitrogen) and a fluorescein isothiocyanate (FITC)-conjugated anti-BrdU antibody (Invitrogen). Apoptotic cells were detected using an Annexin V-FITC Apoptosis Detection Kit (BD Biosciences). The cells were analyzed using BD FACSAria III (BD Biosciences), and data analysis was performed using FlowJo v10.0 (BD Biosciences).

### Human ether-a-go-go related gene (hERG) binding assay and patch-clamp assay

Both hERG assays were conducted as described previously^21^.

### Cell migration and invasion assay

For the cell migration assay, the cells were added to an 8 μm pore transwell polycarbonate chamber (SPL Life Science). After 24 h of incubation, the number of cells that migrated through the membrane into the lower chamber was counted. For the cell invasion assay, Matrigel (dilution at 1:5; BD Biosciences) was added to the Transwell chambers and incubated for 5 h. The cells were then seeded; after 24 h of incubation, the invaded cells on the bottom of the membrane were fixed with 10% formalin for 20 min and stained with 0.1% crystal violet for 3 h. The extent of invasion was expressed as the average number of cells per microscopic field.

### Sphere formation assay

DLD1 cells were cultured in DMEM/F-12 with epidermal growth factor (20 ng/mL), basic fibroblast growth factor (20 ng/mL), and B27 supplements in noncoated dishes. For serial passaging, the sphere cells were dissociated into single cells with Accutase (Invitrogen) once every 4–7 days and incubated. After the secondary generation, the DLD1 sphere cells were used for the experiments. The cells (5 × 10^4^) were cultured in 6-well plates in the absence or presence of A3373 for 4 days. The cells were fixed with 4% paraformaldehyde, and sphere formation was calculated from 10 positions in each well by an Olympus BX53 microscope and cellSens image acquisition software (Olympus).

### Immunoprecipitation and western blotting

As previously described, lysates from cells or colorectal cancer (CRC) tissues were analyzed by immunoprecipitation, and/or Western blotting was performed as described previously^19^. The antibodies used are listed in Supplementary Table 5. A polyclonal anti-PLD antibody that recognizes both PLD1 and PLD2 was generated, as previously described^22^. The use of human CRC tissue samples was approved by the Institutional Review Board of Pusan National University, Korea (PNU IRB/2011-6).

### Measurement of extracellular ATP and HMGB1 levels

For the quantitative determination of extracellular ATP, the cells were treated with Α3373. Extracellular release of ATP was assessed using the ENLITEN ATP Assay kit (Promega) according to the manufacturer’s instructions. For the HMGB1 level measurement, the supernatants of the cells were prepared by removing insoluble materials by centrifugation at 10,000 × *g* for 3 min at 4 °C. The levels of HMGB1 were measured using an HMGB1 detection kit (Chondrex) according to the manufacturer’s instructions.

### In vitro phagocytosis assay

For the phagocytosis assay, MC38 cells were treated with A3373 in DMEM containing 1% FBS for 48 h and were labeled with 2 μM carboxyfluorescein succinimidyl ester (CFSE). A total of 5 × 10^5^ THP1 cells were incubated with equal numbers of CFSE-labeled cells in DMEM without FBS for 2 h in a 24-well plate. The cells were washed with PBS and incubated with human BD Fc block (BD Biosciences) to block nonspecific detection. Furthermore, the cells were incubated with PerCP-Cy5.5-conjugated anti-CD11b (BD Biosciences) for 20 min at 4 °C and were analyzed by flow cytometry.

### Animal experiments

The AOM/DSS CRC, colon cancer orthotropic, and colon cancer syngeneic models were used in this study. The details are described in the Supplementary Materials and Methods section.

### Cytotoxic T-cell-mediated killing assay

GFP-luciferase expressing MC38 murine CRC (MC38-GFP-Luc) cells were cocultured with bone marrow-derived macrophages (BMDMs). After 48 h, the macrophages were isolated and incubated with naïve T cells from the splenocytes of wild-type mice to generate CD8^+^ T cells. The activated CD8^+^ T cells were sorted using BD FACSAria III, added to wells containing MC38-GFP-Luc cells and titrated to effector cells (E) and the target cell (T) ratio (E/T ratio). Four hours later, CD8^+^ T-cell-mediated killing of MC38-GFP-Luc cancer cells in each well was monitored using a luciferase-based bioluminescence imaging system.

### Statistical analysis

The results are expressed as the mean ± standard deviation (SD). The statistical significance of the differences was determined by Student’s *t test*, and *P* < 0.05 was considered statistically significant. Kaplan‒Meier curves were analyzed using the log-rank test. Statistical analysis was performed using Prism 8.0 for Windows (GraphPad).

## Results

### Development of a potent PLD1 inhibitor based on computer-aided drug design and pharmacophore study

**Fig. 1:**
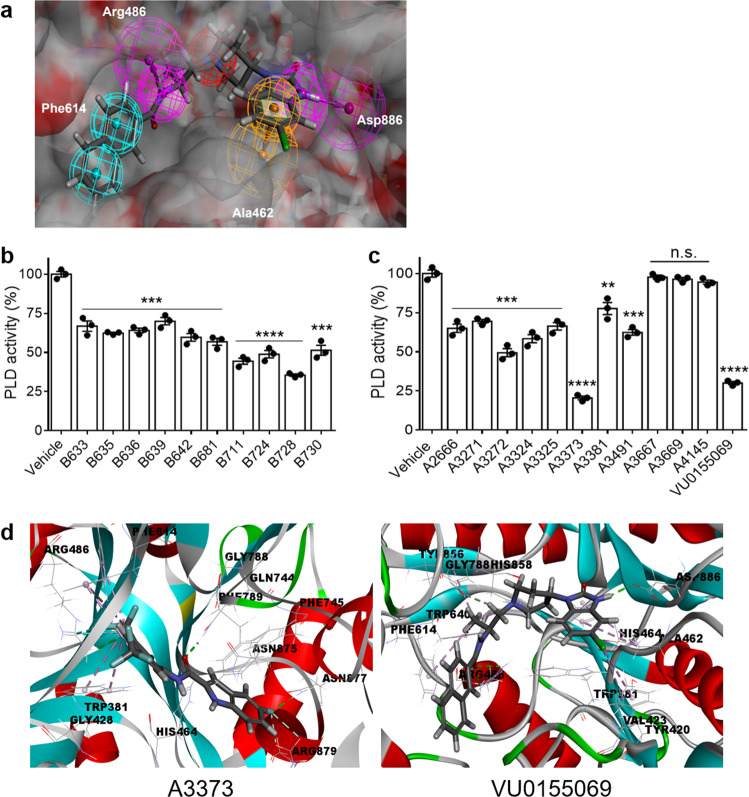
Development of a potent PLD inhibitor based on computer-aided drug design and pharmacophore studies. **a** 3D structure-based pharmacophore model of VU0155069 in complex with PLD1 based on the X-ray crystal structure of PLD1 (PDB ID: 6OHR). The pharmacophore features were generated after the docking simulation of PLD1 with VU0155069. **b** Effect of various compounds selected in the virtual screening process on PLD activity in HCT 116 cells. **c** Synthetic compounds derived from B728 were tested for PLD activity. **d** Binding mode of A3373 or VU0155069 to PLD1. The binding mode of A3373 docked on PLD1 is shown as a stick model (green: hydrogen bond, pale pink: π-alkyl interaction, and carbon-halogen: cyan). The results are representative of at least three independent experiments and presented as the mean ± SD. **P < 0.01; ***P < 0.001; ****P < 0.0001, n.s. not significant.

To develop improved PLD inhibitors, we attempted to obtain a structure-based pharmacophore corresponding to the catalytic pocket of PLD1 by docking VU0155069, a known PLD1 inhibitor^2^, to the crystal structure of hPLD1 (PDB: 6OHR). Several pharmacophore features were generated based on the inhibitor-PLD1 complex interaction (Fig. 1a), which showed that hydrophobic interactions were formed with the Phe614 residue of the protein, and ring aromatic features were formed with Ala462. Hydrogen bond donor features were found in the interactions with Arg486 and Asp886 residues of the protein, whereas the nitrogen atoms in the piperidine ring showed positive ionizable features (Fig. 1a). These pharmacophore interaction features were applied to 3200 commercial compounds from the in-house library. The fitness value of each compound was evaluated using a virtual screening process based on the pharmacophores of VU0155069 in the complex with PLD1. FitValue is a measure of how well the ligand fits the pharmacophore. The higher the fit score is, the better the match. Ten compounds with a FitValue ranging from 2.5 to 3.5 were generated and evaluated for the inhibitory activity of PLD in HCT 116 CRC cells (Fig. 1b and Supplementary Table 1). Three compounds (B711, B724, and B728**)** showed PLD activity that was less than 50% of the total activity, and B728 suppressed the most potent PLD activity (Fig. 1b). These compounds contain an indole moiety, which might be involved in the inhibition of PLD. Therefore, we designed a new series of PLD inhibitors comprising an indole moiety. Based on the structure of B728, the synthesized compounds contained an amide group as a linker and an aromatic ring to introduce diverse substituents. Eleven compounds were synthesized and evaluated for their PLD activity (Fig. 1c and Supplementary Table 2). Among these, A3373 showed the most potent inhibition of PLD activity (20.6% of the activity remained at 10 μM) compared with that of VU0155069 as a reference (30.4% of the activity remained at 10 μM). Next, we analyzed the binding of A3373 and VU0155069 with PLD1 and PLD2. As shown in Fig. 1d, in the docking model of A3373 and PLD1, the amide linker of A3373 underwent hydrogen bonding to the Gln744 side chain of PLD1. A3373 showed a reasonable binding energy in PLD1 (−193.46 kcal/mol) (Supplementary Table 3). In contrast, the amide linker of A3373 did not have a hydrogen bond with any amino acid of PLD2, which was different from the A3373–PLD1 binding model (Supplementary Fig. 1). In addition, the CF_3_ group of A3373 interacted with several amino acids of PLD2, similar to the binding model with PLD1, suggesting that the CF_3_ group might play a role in the interaction between A3373 and PLD isozymes. Moreover, the binding model of VU0155069 with PLD2 showed a more stable binding energy (−192.83 kcal/mol) than that of A3373 with PLD2 (−151.13 kcal/mol), whereas both drugs exhibited similar binding energies in PLD1 (Supplementary Table 3). Therefore, it can be assumed that A3373 might display more potent binding to the catalytic pocket of PLD1 than to that of PLD2. Collectively, these data suggest that a potent PLD inhibitor can be developed based on computer-aided drug design (CADD) and pharmacophore studies.

### A3373 selectively inhibits PLD1 activity via direct binding with PLD1

**Fig. 2:**
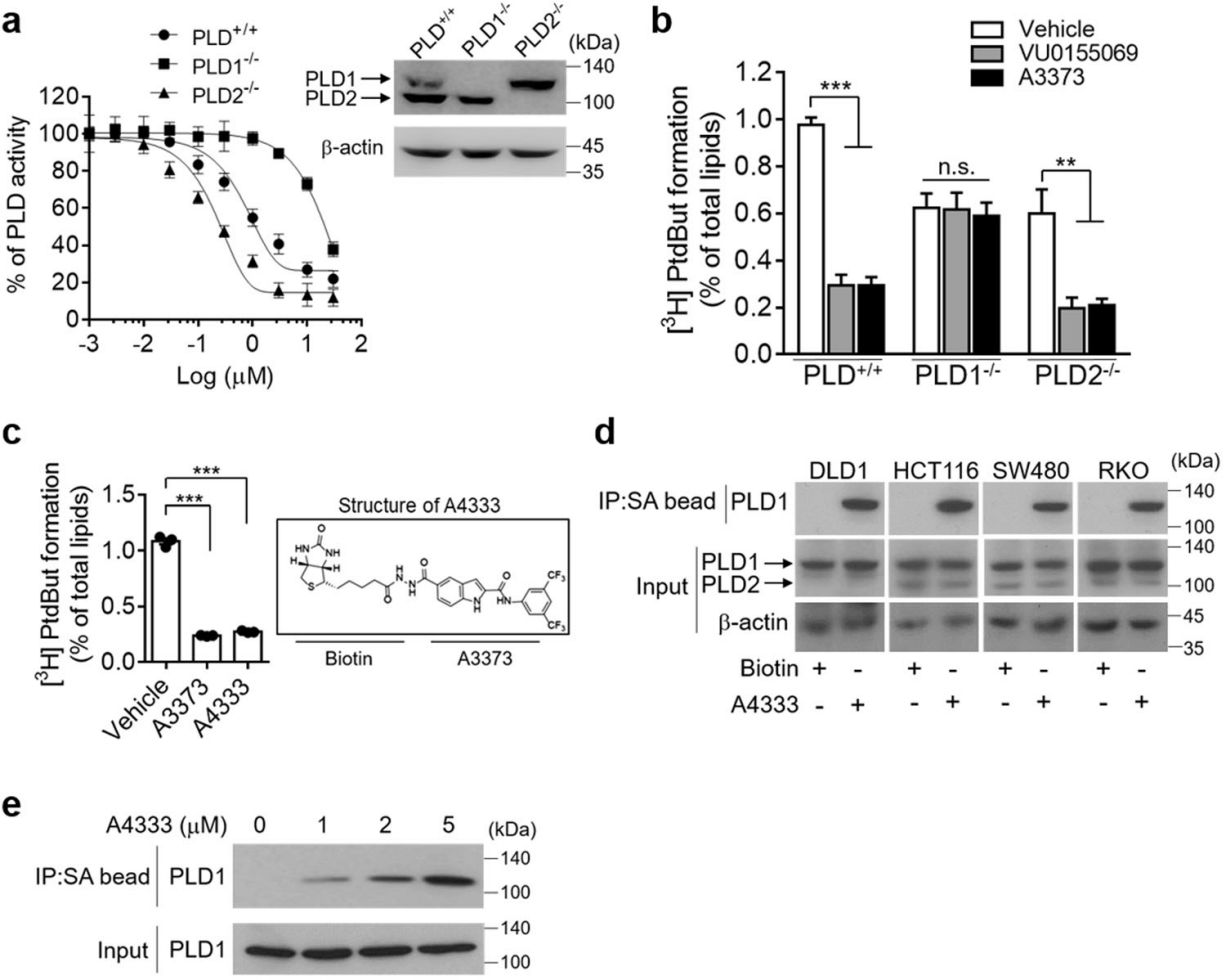
A3373 selectively inhibits PLD1 activity via direct binding. **a** IC_50_ for inhibition of PLD activity by A3373 was determined in the indicated *Pld*^*−/−*^ MEFs. PLD expression was analyzed by immunoblotting. **b** The *Pld*^*−/−*^ MEFs were treated with 2 μM of the indicated PLD inhibitors, and PLD activity was determined. **c** Effect of biotinylated A3373 on PLD activity in DLD1 cells. **d** Effect of biotinylated A3373 on the binding of PLD isozymes in the indicated CRC cells, as analyzed by a streptavidin pull-down assay and immunoblotting. **e** In vitro binding assay of A4333 with in vitro-translated PLD1. The results are representative of at least three independent experiments and presented as the mean ± SD. **P < 0.01; ***P < 0.001, n.s not significant.

We further investigated the effect of A3373 on the enzymatic inhibition of PLD1 and PLD2 in *Pld*^*+/+*^, *Pld1*^*−/−*^, and *Pld2*^*−/−*^ MEFs. The *Pld1*^*−/−*^ and *Pld2*^*−/−*^ MEFs expressed PLD2 and PLD1 proteins, respectively, as confirmed using PLD antibody, which recognizes both PLD1 and PLD2 (Fig. 2a). A3373 showed more potent inhibition of PLD1 than PLD2; the IC_50_ for PLD1 activity was 325 nM, and the IC_50_ for PLD2 activity was 15.15 μM. (Fig. 2a). PLD1 activity, but not PLD2 activity, was suppressed using 2 μM A3373 and VU0155069 (Fig. 2b). Moreover, the drugs significantly suppressed PLD activity in PLD1-overexpressing HCT116 cells but not in PLD2-overexpressing cells (Supplementary Fig. 2a). These data suggested that A3373 selectively inhibited PLD1 activity. Kinase profiling was used to evaluate the general selectivity of the lead compounds. A3373 produced no effect on the activity of the 21 purified protein kinases (Supplementary Fig. 2b). To further investigate whether inhibition of PLD1 by A3373 occurs by binding to PLD1, we first modified A3373 by adding biotinylation instead of the F present in its structure and measured PLD activity. Compared to the unsubstituted congener, biotinylated A3373 (A4333) was still potent for PLD inhibition (Fig. 2c). Using a streptavidin pull-down assay, we found that A4333 interacted with PLD1, but not PLD2, in various CRC cells (Fig. 2d). We further validated that A3373 directly interacts with the in vitro-translated PLD1 protein (Fig. 2e). Collectively, these results suggest that A3373 may be a selective inhibitor of PLD1 via its binding to PLD1.

### A3373 regulates the proliferation and apoptosis of CRC cells but not normal colonic cells, exhibits no in vitro toxicity, and is an effective orally administered compound

**Fig. 3:**
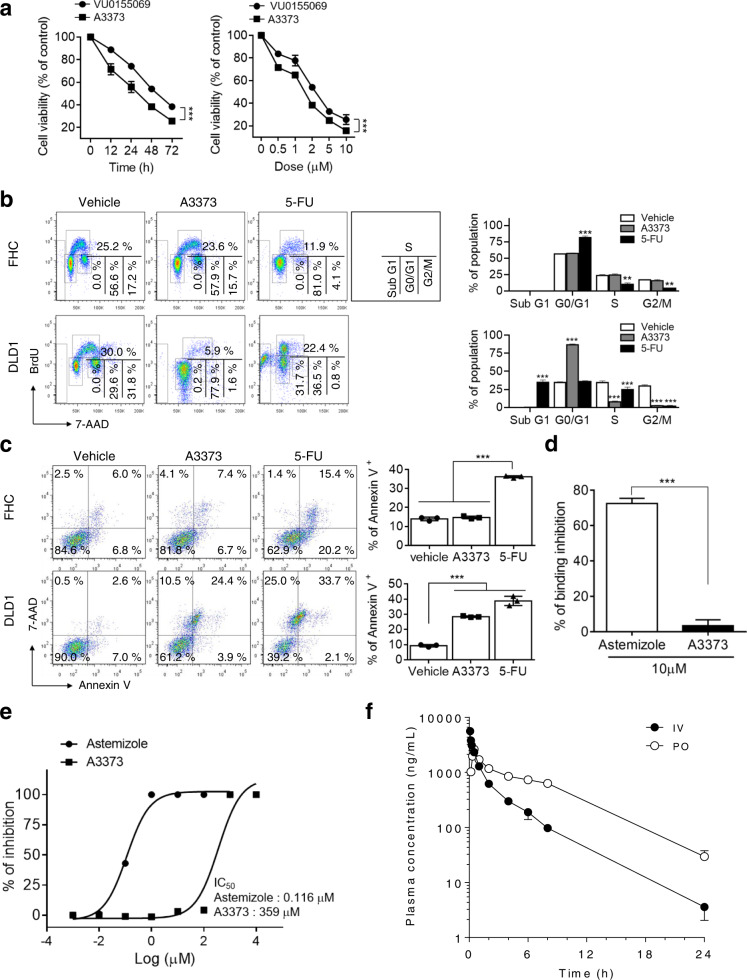
PLD1 inhibition suppresses CRC cell proliferation but promotes apoptosis and is safe in vitro with no toxicity. **a** Cell viability was examined in DLD1 cells treated with PLD inhibitors in a time-dependent (for 48 h) and dose-dependent (at 2 μM) manner. **b** A3373 (2 μM) and 5-FU (10 μM) treatment was administered to the indicated cells, and the cell cycle was analyzed. The population at the indicated cell cycle phase was quantitated. **c** CRC cells were treated with the indicated drugs for 48 h, and apoptosis was analyzed in the presence of 1% FBS using annexin V and flow cytometry. **d**, **e** hERG ligand-binding assay (**d**) and patch-clamp assay (**e**) in HEK293 cells transfected with hERG. **f** Plasma concentration-time curves of A3373. Single-dose plasma pharmacokinetics of A3373 in male ICR mice following 5 mg/kg (intravenous injection, IV) or 10 mg/kg (peroral injection, PO). Data are presented as the mean (*n* = 3). The results are representative of at least three independent experiments and presented as the mean ± SD. **P < 0.01; ***P < 0.001.

We further investigated the effect of the drugs on the viability of CRC cells. A3373 and VU0155069 significantly inhibited the viability of DLD1 cells in a dose- and time-dependent manner, in which A3373 showed a higher potency in suppressing viability than that of VU0155069 (Fig. 3a). We further examined whether A3373 affects the cell cycle. 5-Fluorouracil (5-FU), an anticancer drug used in the clinic, decreased the S phase population in FHC, normal fetal colonic mucosa cells, and CRC cells, as analyzed by flow cytometry using BrdU (Fig. 3b and Supplementary Fig. 3a). However, A3373 had no effect on FHC cells but significantly decreased the proliferation of CRC cells (Fig. 3b and Supplementary Fig. 3a). Moreover, 5-FU increased the apoptosis of both CRC and FHC cells, whereas A3373 significantly promoted the apoptosis of CRC cells alone (Fig. 3c and Supplementary Fig. 3b). Thus, A3373 might have a selective effect on CRC cells but not on normal colonic cells. To further verify the safety of A3373, the potential cardiac-associated side effects of A3373 were evaluated in vitro using an hERG ligand-binding assay and patch-clamp assay in HEK293 cells transfected with hERG. The hERG channel is a voltage-gated potassium channel that is known for its contribution to the electrical activity of the heart, which helps coordinate the heartbeat^23^. Thus, an important factor for drug development is the lack of hERG inhibition. At 10 μM, astemizole, a positive control, showed an inhibition of 72.5% in the ligand-binding assay and 0.116 μM IC_50_ in the patch-clamp assay (Fig. 3d, e). However, A3373 did not interact with the hERG channel (3.4% inhibition at 10 μM) or inhibit the hERG K^+^ channel (IC_50_ > 50 μM) (Fig. 3d, e). Furthermore, the in vivo pharmacokinetics of Compound A3373 were tested. Figure 3f shows the mean plasma concentration-time curves for A3373 after intravenous (IV) and oral administration. The corresponding pharmacokinetic parameters are summarized in Supplementary Table 4. A3373 was administered intravenously and orally at 5 and 10 mg/kg, respectively, to male ICR mice. The plasma concentration profile of A3373 showed a peak plasma concentration of C_max_ (2629.6 ± 182.6 ng/mL) and T_max_ (30 min) with oral administration. The AUC_inf_ (Area Under the Curve) values of A3373 from the intravenous and oral administrations were 6316.8 ± 363.7 ng/h/mL and 13,647.5 ± 1506.9 ng/h/mL, respectively. The clearance and steady state volume of A3373 during intravenous administration were 0.8 ± 0.1 L/h/kg and 2.1 ± 0.1 L/kg, respectively. The terminal half-lives of A3373 were ~3.2 h and 3.8 h after intravenous and oral dosing, respectively. The oral bioavailability for A3373 was 108.0%. Collectively, these results suggest that A3373 selectively suppresses proliferation, promotes apoptosis in CRC cells but not normal cells, shows no in vitro cardiotoxicity and exhibits an effectiveness in oral administration.

### A3373 suppresses Wnt/β-catenin signaling and inhibits migration, invasion and self-renewal capacity in CRC cells

**Fig. 4:**
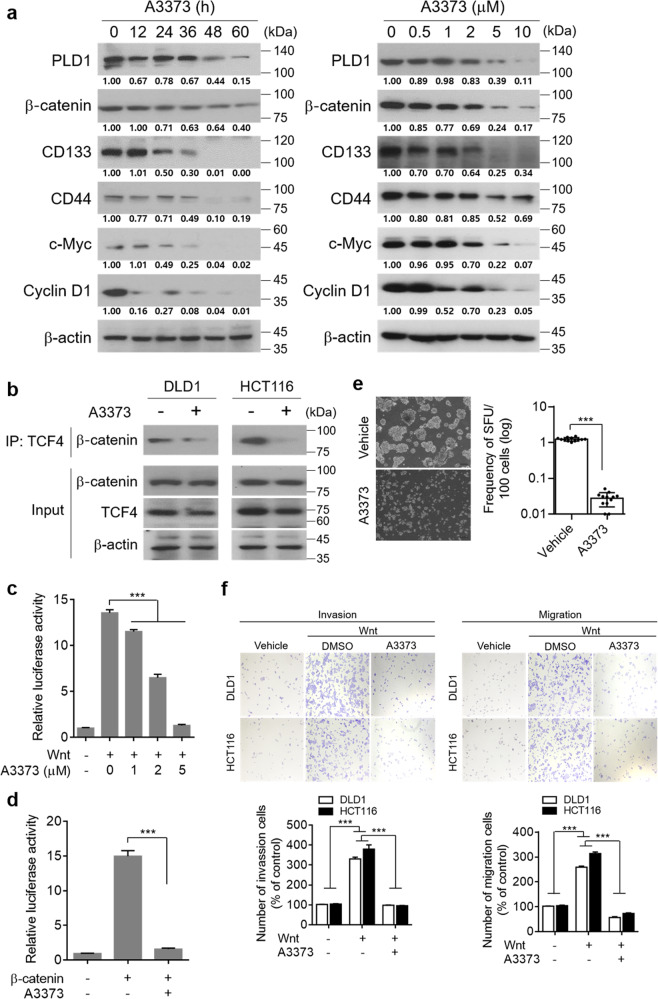
PLD1 inhibition reduces Wnt/β-catenin signaling and suppresses migration, invasion and self-renewal capacity. **a** HCT 116 cells were treated with A3373 in a time- (for 36 h) and dose-dependent (at 2 μM) manner in the presence of 1% serum. The lysates were analyzed by immunoblotting. **b** CRC cells were treated with A3373 (2 μM) for 12 h, and the lysates were immunoprecipitated and/or immunoblotted with the indicated antibodies. **c** Effect of A3373 on Wnt3a (150 ng/mL)-induced TCF transactivation in HEK293 cells. **d** Effect of A3373 on β-catenin-mediated TCF transactivation in HEK293 cells. **e** Effect of A3373 on the sphere-forming capacity of DLD1 cells. Limiting dilution assays were performed by diluting sphere-cultured cells. **f** Effect of A3373 on Wnt3a-induced migration and invasion. The results are representative of at least three independent experiments and presented as the mean ± SD. ***P < 0.001.

Abnormal activation of the Wnt/β-catenin pathway is responsible for the initiation and progression of almost all CRCs^24^. Previously, we reported that PLD1 is a transcriptional target gene of β-catenin/TCF and promotes Wnt/β-catenin signaling^5,6,20^. Thus, we investigated whether A3373 affected the expression of β-catenin and its downstream target genes. A3373 greatly reduced the protein levels of β-catenin and its target genes, including PLD1, CD133, CD44, c-Myc, and cyclin D1, in a time- and dose-dependent manner (Fig. 4a). Furthermore, we examined the mechanistic role of A3373 in regulating β-catenin expression. Recently, we reported that the 3′ untranslated region (UTR) of β-catenin contains a miR-4496 binding site^20^. A3373 increased the promoter activity of miR-4496 (Supplementary Fig. 4a) and significantly suppressed the luciferase activity of the β-catenin 3′-UTR reporter in HCT116 cells (Supplementary Fig. 4b), suggesting that the inhibition of PLD1 occurs by A3373 downregulates β-catenin via the upregulation of miR-4496. Because the integrity of the β-catenin/TCF complex is necessary for proper transcriptional activity, we evaluated the effect of A3373 on the interaction between β-catenin and TCF-4. A3373 treatment (2 μM) for 12 h disrupted the endogenous interaction between β-catenin and TCF-4, whereas the levels of β-catenin and TCF-4 were not affected (Fig. 4b). Recently, we reported that PLD1 inhibition reduced the binding of β-catenin to TCF4 by increasing the association of ICAT with β-catenin and upregulating ICAT, a negative regulator of Wnt signaling that is known to competitively bind to β-catenin to inhibit β-catenin/TCF-4 complex formation^25,26^. Thus, it is suggested that pharmacological inhibition of PLD1 by A3373 might decrease the interaction of β-catenin with TCF4, probably via upregulation of ICAT and its competitive binding to β-catenin. A3373 significantly decreased Wnt3a- and β-catenin-induced TCF transactivation in HEK293 cells with wild-type APC and β-catenin (Fig. 4c, d). Moreover, A3373 significantly suppressed TCF transactivation in CRC cells with an active Wnt pathway (Supplementary Fig. 4c). The Wnt/β-catenin pathway is a major regulator of colon cancer-initiating cell (CIC) genesis and the self-renewal of normal cells^24^. Treatment with A3373 in DLD1 cells significantly decreased the expression of CD44 and CD133, markers of CIC, in a dose-dependent manner, as assessed by the mean fluorescence intensity using flow cytometry (Supplementary Fig. 4d). A3373 significantly decreased the sphere-forming capacity of DLD1 cells, as analyzed with limiting dilution assays (Fig. 4e). Additionally, A3373 significantly suppressed Wnt3a-induced migration and invasion of CRC cells (Fig. 4f). These results suggest that inhibition of PLD1 by A3373 suppresses the migration, invasion, and self-renewal capacity of CRC cells by downregulating the Wnt/β-catenin signaling pathway.

### Pharmacological inhibition of PLD1 downregulates “do not eat me” signals and upregulates “eat me” signals, subsequently enhancing phagocytic activities

**Fig. 5:**
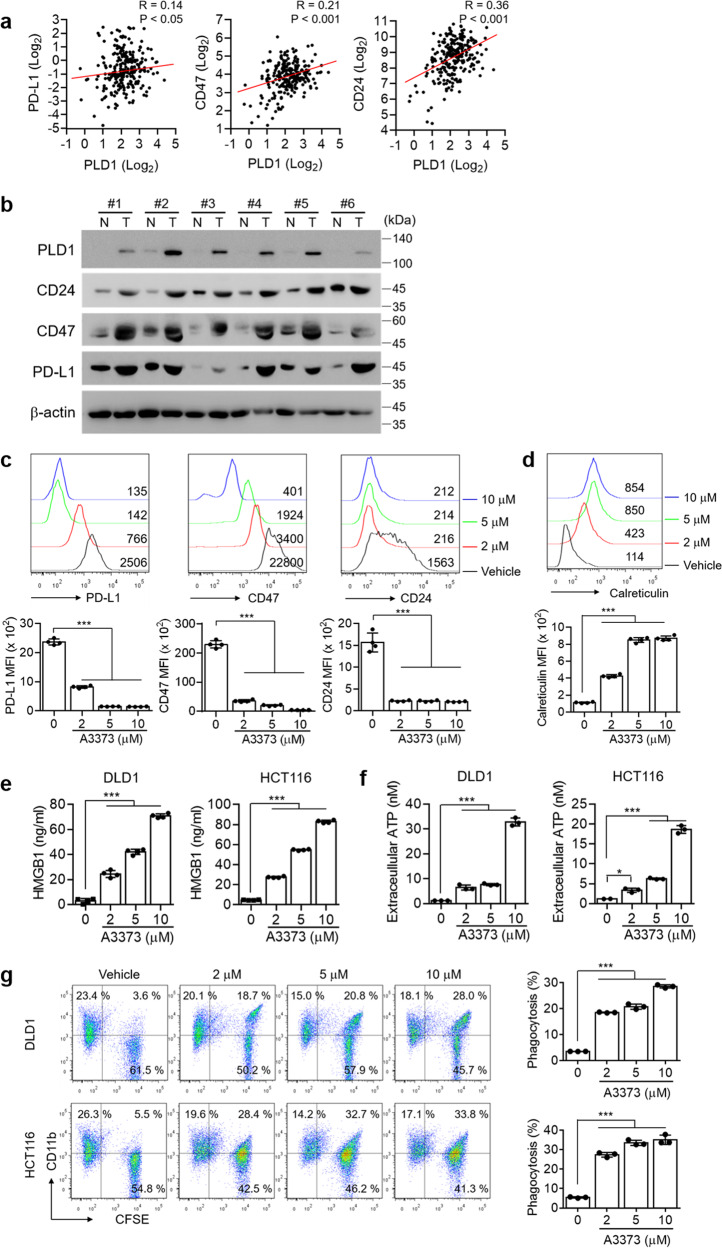
PLD1 inhibition in CRC cells enhances phagocytic activities by downregulating “do not eat-me” signals and upregulating ‘eat-me’ signals. **a** PLD1 expression is positively correlated with the level of the indicated “do not eat-me” signals in CRC as analyzed by TCGA database. **b** The lysates of CRC tissues were analyzed by immune blotting using the indicated antibodies. N adjacent nontumor, T tumor. **c** Effect of A3373 on the levels of the “do not eat-me” signals in HCT116 cells as analyzed by flow cytometry. **d** Effect of A3373 on the level of calreticulin in HCT116 cells. **e** Effect of A3373 on the amount of secreted HMGB1 as analyzed by ELISA. **f** Effect of A3373 on the level of extracellular ATP. **g** Effect of A3373 on the phagocytic activity of macrophages. Right, the population of CFSE^+^CD11b^+^ macrophages was quantified. The results are representative of at least three independent experiments and presented as the means ± SDs. ***P < 0.001.

The “do not eat-me” signals, including CD47, CD24 and programmed cell death ligand 1 (PD-L1), result in tumors that prevent macrophage surveillance and clearance^27–29^. Mining the Cancer Genome Atlas (TCGA) database revealed that the expression of PLD1, but not PLD2, was positively correlated with the expression of PD-L1, CD47, and CD24 in CRC tumors (Fig. 5a and Supplementary Fig. 5a). Moreover, the expression of PLD1 and the “do not eat-me” signals was upregulated in CRC tumors compared with normal colon tissues, as analyzed by immunoblotting (Fig. 5b). Interestingly, A3373 significantly reduced the levels of the “do not eat-me” signals in CRC cells, as analyzed by flow cytometry, whereas overexpression of PLD1 increased their levels (Fig. 5c and Supplementary Fig. 5b, c). “Eat-me” signals, such as CRT on the apoptotic cell surface, are sensed by phagocytes to promote engulfment^30,31^. Furthermore, phagocytosis of cells expressing CRT on their surface appears to induce immunogenic responses^32^. Treatment with A3373 increased cell-surface CRT expression (Fig. 5d and Supplementary Fig. 5d). HMGB1 is released into the extracellular environment during cell death and alerts the innate immune system by acting as a chemoattractant for inflammatory leukocytes, triggering the activation of phagocytes^33^. A3373 increased the release of HMGB1 in a dose-dependent manner (Fig. 5e). ATP is a critical and nonredundant “find me” signal released by apoptotic cells^34^. A3373 significantly increased extracellular ATP (Fig. 5f). Furthermore, the phagocytic activity of macrophages was assessed on A3373-treated CFSE-labeled CRC cells cocultured with phorbol 12-myristate 13-acetate (PMA)-activated THP1 macrophages, which showed that A3373 significantly increased the population of CD11b^+^CFSE^+^ macrophages compared to that in vehicle-treated cells (Fig. 5g). Collectively, these findings suggested that pharmacological inhibition of PLD1 enhanced the phagocytic activities of cancer cells by macrophages via modulation of ‘do not eat-me’ and ‘eat-me’ signals.

### A3373 attenuates colitis-associated CRC and modulates Wnt signaling, phagocytosis checkpoints and immune signaling

**Fig. 6:**
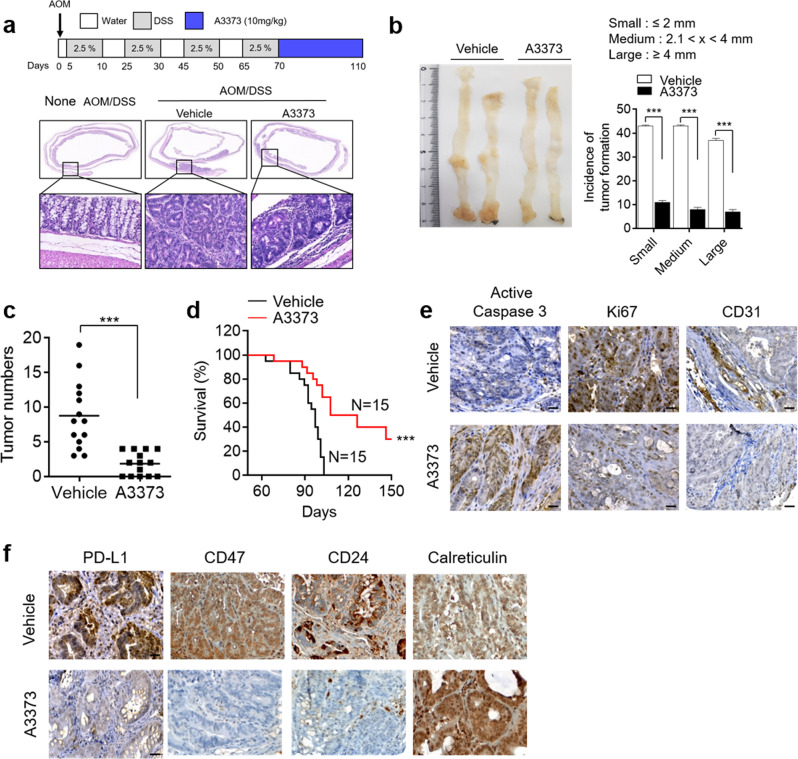
PLD1 inhibition attenuates tumor formation, downregulates ‘do not eat-me’ signals and upregulates ‘eat-me’ signals in colitis-associated CRC. **a** Schematic view of the colitis-associated cancer model (top). AOM/DSS-treated mice were treated with either vehicle or A3373 (10 mg/kg) three times a week for 6 weeks, and AOM/DSS-vehicle (*n* = 12) or AOM/DSS*-A3373 (n* = 12) mice were sacrificed at 110 days. H&E staining of the entire Swiss roll of the colon intestine (bottom). **b**, **c** The number of visible polyps in the colon intestine. **d** The indicated mice were followed for long-term survival. The survival probability was analyzed using Kaplan–Meier analysis. **e** IHC for active caspase-3, Ki67 and CD31 in tumor tissues from vehicle- or A3373-treated mice. Scale bar, 20 µm. **f** IHC of the indicated phagocytosis checkpoints in the tumor tissues. Scale bar, 20 µm. Representative images were selected from at least six different fields. ***P < 0.001.

We further investigated whether A3373 inhibited colitis-associated CRC using an AOM/DSS-induced mouse colon cancer model^35^. A3373 attenuated the tumor formation in the AOM/DSS-induced mice (Fig. 6a), and the polyps in the large intestine were smaller than those in the control mice (Fig. 6b). The number of intestinal polyps was also significantly lower than that in AOM/DSS control mice (Fig. 6c). In addition, mortality in A3373-treated AOM/DSS mice was significantly lower than that in AOM/DSS control mice (Fig. 6d). A3373 increased the level of active caspase-3 and decreased the levels of Ki67 and CD31, suggesting that A3373 enhances apoptosis and reduces both proliferation and tumor angiogenesis in cancer cells (Fig. 6e). Furthermore, treatment with A3373 in AOM/DSS mice decreased the expression of “do not eat-me” signals but increased that of “eat-me” signals (Fig. 6f). Moreover, A3373 suppressed the levels of β-catenin and its downstream target proteins, as analyzed by IHC (Supplementary Fig. 6a). A3373 also reduced the expression of immune checkpoints (PD1 and B7H4) and Foxp3, a marker of regulatory T cells, but increased the expression level of CD8, a marker of cytotoxic T cells, as analyzed by IHC (Supplementary Fig. 6b). Collectively, these results suggest that the inhibition of PLD1 attenuates colitis-associated CRC, probably by controlling multiple pathways, including Wnt signaling, phagocytosis checkpoints, and immune signaling.

### A3373 renders cancer cells more susceptible to cytotoxic CD8^+^ T-cell-mediated killing via the increased phagocytic activity of macrophages

**Fig. 7:**
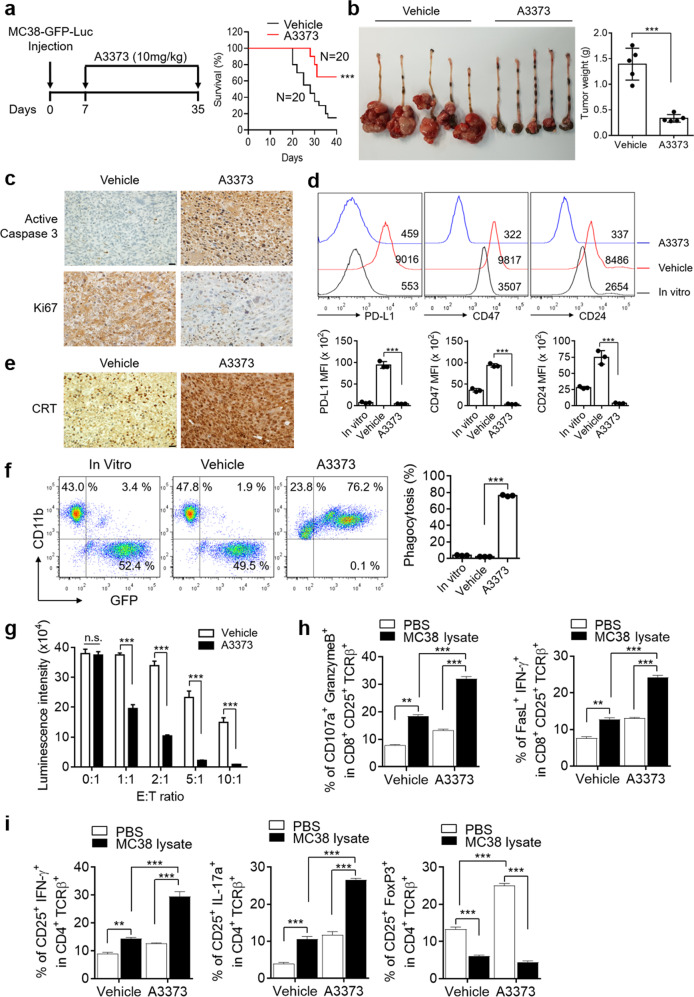
Increased phagocytic activity of macrophages mediated by cytotoxic CD8+ T-cell-dependent killing of cancer cells in PLD1 inhibition. **a** MC38-GFP-Luc cells were orthotopically injected into the cecal submucosa of mice and then treated with A3373 (10 mg/kg) every day for 4 weeks. The mortality of tumor-bearing mice was analyzed using Kaplan–Meier analysis. **b** The tumors that developed at the injection site were photographed by an optical camera, and their weight was quantified. **c** IHC of active caspase-3 and Ki67 in the tumor tissues. Scale bar, 20 µm. **d** The levels of “do not eat-me” signals in the cancer cells isolated from vehicle- or A3373-treated tumor-bearing mice were analyzed by flow cytometry. **e** IHC of CRT in the tumor tissues. Scale bar, 20 µm. **f** The MC38-GFP-Luc cells derived from A3373-treated mice were cocultured with BMDMs for 2 h, and phagocytosis activity was analyzed by flow cytometry. The population of GFP^+^CD11b^+^ macrophages was quantified. **g** Cytotoxic T-cell-mediated killing assay of MC38-GFP-Luc cells derived from orthotopically injected tumors. **h** The populations of CD107a^+^ granzyme B^+^ and FasL^+^IFNγ^+^ in activated CD8^+^ T cells were analyzed by flow cytometry. **i** IFNγ^+^, IL17a^+^, and Foxp3^+^ cells in activated CD4^+^ T cells were analyzed by flow cytometry. The results are representative of at least three independent experiments and presented as the means ± SDs. **P < 0.01; ***P < 0.001, n.s. not significant.

Next, the effect of A3373 on tumor development was assessed using MC38-GFP-Luc cells that were orthotopically injected into the cecal submucosa of mice. Daily A3373 treatment led to a significant reduction in mortality and tumor weight compared with that of the vehicle treatment (Fig. 7a, b). Moreover, treatment with A3373 resulted in increased levels of active caspase-3 and reduced levels of Ki67 in tumor tissues (Fig. 7c). The cancer cells derived from tumor-bearing mice showed higher levels of the “do not eat-me” signals compared with those in the MC38 cells cultured in vitro, whereas treatment with A3373 showed downregulation of “do not eat-me” signals (Fig. 7d and Supplementary Fig. 7a). Moreover, A3373 increased the expression of the “eat-me” signal and CRT (Fig. 7e) and significantly reduced the levels of β-catenin and PLD1 (Supplementary Fig. 7b). Furthermore, in the cancer cells from A3373-treated tumor-bearing mice, downregulation of immune checkpoints and cancer stem cell markers were observed (Supplementary Fig. 7c, d). The level of H-2^b^ (MHC-I in humans) is reduced in tumors for immune evasion. The level of H-2^b^ was significantly increased in the tumors from the A3373-treated group compared to those from the vehicle group (Supplementary Fig. 7c). We further investigated whether macrophages increased the phagocytosis of cancer cells in A3373-treated tumor-bearing mice. Here, the phagocytic activity of BMDMs was assessed in a coculture assay with MC38-GFP-Luc cells isolated from tumors by flow cytometry. Treatment with A3373 significantly increased the population of GFP^+^CD11b^+^ BMDMs, suggesting that the phagocytosis of MC38-GFP-Luc cancer cells derived from A3373-treated mice was enhanced (Fig. 7f). We further investigated whether the treatment of MC38 cells with A3373 renders cancer cells more susceptible to MC38-specific T-cell-mediated killing. CD8^+^ T-cell-mediated killing of MC38-GFP-Luc cells was monitored using a luciferase-based bioluminescence imaging system. Cancer cells incubated with vehicle alone showed a significant reduction in luciferase activity as the effector cell (E)/target cell (T) ratio increased (Fig. 7g). MC38-GFP-Luc cells derived from A3373-treated mice showed significantly lower luciferase activity than those derived from vehicle-treated tumor-bearing mice (Fig. 7g). These data suggested that the increased tumor lysis by A3373 might be due to enhanced antitumor immunity via activated CD8^+^ cytotoxic T cells. Next, we investigated whether A3373 affects anticancer efficacy via the activation of CD8^+^ T cells. Mesenteric lymph nodes from orthotopically injected mice were isolated and incubated with lysates of MC38-GFP-Luc cells in vitro to restimulate T cells. The mesenteric lymph nodes from A3373-treated mice showed an increased population of CD107a^+^ granzyme B^+^ and FasL^+^IFNγ^+^ CD8^+^ T cells in activated CD8^+^ T cells compared with that of the vehicle-treated mice (Fig. 7h and Supplementary Fig. 7e). Furthermore, treatment with A3373 significantly increased the population of IFNγ^+^ and IL17a^+^ in CD4^+^ T cells, whereas the mesentric lymph nodes from A3373-treated mice showed a decreased population of Foxp3^+^ cells in the CD4^+^ TCRβ^+^ gate compared with that of vehicle-treated mice (Fig. 7i and Supplementary Fig. 7f). Collectively, these results suggest that A3373 might exert anticancer activity via the upregulation of activated cytotoxic CD8^+^ T cells and Th17 cells, as well as downregulation of the Treg cell-mediated immune response. Taken together, these data suggested that A3373 may render MC38 cells more susceptible to MC38-specific T-cell-mediated killing and might exert anticancer activity via the upregulation of activated cytotoxic CD8^+^ T cells and Th17 cells, as well as the downregulation of immune response regulated by Treg cells.

### Combination therapy with A3373 and an anti-PD-L1 antibody further enhances tumor regression via immune activation in the tumor environment

**Fig. 8:**
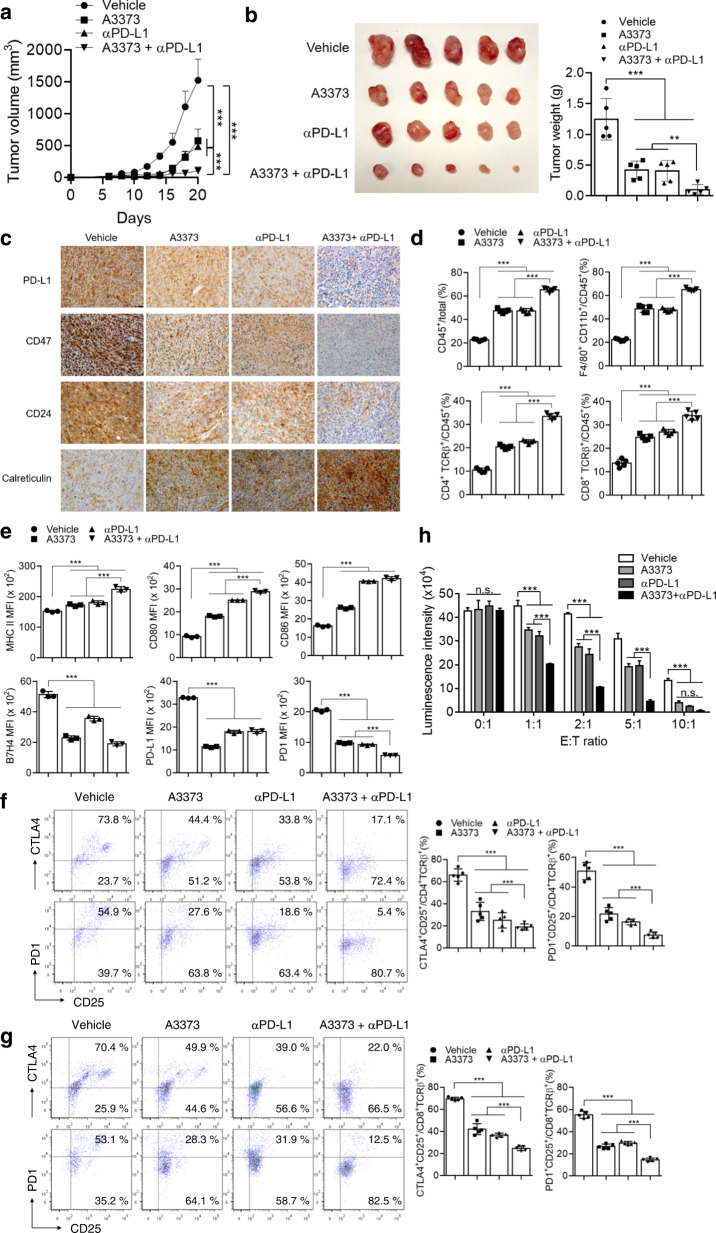
Enhanced tumor regression in a combination therapy of PLD1 inhibitor and αPD-L1 antibody via immune activation. **a** Syngeneic mice were subcutaneously injected with MC38-GFP-Luc cells and treated with A3373 (10 mg/kg) and/or αPD-L1 (100 μg/mouse) at once every 2 days. The tumor volume was measured during the indicated period. **b** Effect of the indicated drug(s) on the tumor weight. **c** Effect of the indicated drug(s) on the expression of “do not eat-me” and “eat-me” signals as analyzed by IHC. Scale bar, 20 µm. **d** Effect of the indicated drug(s) on the population of immune cells that infiltrated into tumors, as analyzed by flow cytometry. **e** Effect of the indicated drug(s) on the phenotyping of macrophages infiltrated into tumors. **f** Effect of the drug(s) on the population of PD1^+^ and CTLA^+^ CD8^+^ T cells infiltrated into tumors. **g** Effect of the drug(s) on the population of PD1^+^ and CTLA^+^ CD4^+^ T cells infiltrated into tumors. **h** Effect of the drug(s) on the CD8^+^ T-cell-mediated killing of MC38-GFP-Luc cancer cells. The results are representative of at least three independent experiments and presented as the means ± SDs. **P < 0.01; ***P < 0.001, n.s. not significant.

To further investigate how A3373 affects antitumor activity with mediation by an anti-PD-L1 antibody (αPD-L1), we used the MC38 syngeneic tumor model. Syngeneic mice were subcutaneously injected with MC38 cells and treated with A3373 and/or αPD-L1 once every 2 days. Either treatment significantly decreased tumor formation, in which further reduction was observed in the combined treatment (Fig. 8a, b). Histological analysis showed that either treatment reduced the expression of “do not eat-me” signals and increased the level of CRT, whereas cotreatment further downregulated and upregulated the “do not eat-me” signals and CRT, respectively, compared with those of the single treatments (Fig. 8c). The expression of Foxp3 was comparable to that of the “do not eat-me” signals (Supplementary Fig. 8a). Moreover, the combined treatment further decreased the expression of β-catenin and Ki67 and increased the levels of active caspase-3 compared to those of the single treatments (Supplementary Fig. 8b). Next, we analyzed the immune cell infiltrates of tumors collected at their maximum volume (1 cm^3^) using flow cytometry. As shown in Fig. 8d, the percentage of immune cells (CD45^+^ cells) in total cells (1 × 10^4^ cells) was higher in either the A3373 or αPD-L1 treatment groups than in the vehicle group (~20% versus 50%). Combinational treatment further increased the percentage of CD45^+^ immune cells (Fig. 8d). Moreover, both treatments significantly increased the percentage of macrophages (F4/80^+^/CD11b^+^), helper T cells (CD4^+^TCRβ^+^), and cytotoxic T cells (CD8^+^TCRβ^+^) among CD45^+^ cells, which were further increased by the combination treatment (Fig. 8d). We further examined the phenotype of macrophages infiltrating the tumors from the drug-treated mice. In macrophages sorted by flow cytometry, A3373 and αPD-L1 significantly increased the expression of MHC-II and costimulatory molecules (CD80 and CD86), which are involved in the activation of T cells (Fig. 8e and Supplementary Fig. 8c), and cotreatment led to further increases in their expression (Fig. 8e). Moreover, both drugs significantly decreased the expression of coinhibitory molecules (B7H4, PD-L1, and PD1), whereas the combined treatment led to a further reduction in B7H4 and PD1 expression but not PD-L1 expression (Fig. 8e and Supplementary Fig. 8c). The expression of PD1 in tumor-associated macrophages negatively correlates with phagocytic potency against tumor cells^29^. We further analyzed the immune checkpoint-expressing T cells that infiltrated the tumors. Both treatments significantly reduced the percentage of PD1^+^/CD25^+^ and CTLA^+^/CD25^+^ T cells among CD4^+^ TCRβ^+^ and CD8^+^ TCRβ^+^ cells, whereas the combined treatment further decreased the percentage of CD4^+^ and CD8^+^ T cells that expressed the immune checkpoints (Fig. 8f, g). These data suggested that combined therapy with A3373 and αPD-L1 may attenuate tumor progression by reducing immunosuppressive PD1 and CTLA signaling to achieve maximal immune activation. We further examined the effect of cotreatment on antitumor immunity via activated CD8^+^ cytotoxic T cells. Splenocytes from tumor-bearing mice were isolated and restimulated with MC38 lysates for 2 days, and the sorted cytotoxic T cells were cocultured with MC38-GFP-Luc cells and titrated to the E/T ratio. The MC38-GFP-Luc cancer cells incubated with CD8^+^ T cells from A3373- or αPD-L1-treated tumor-bearing mice showed a significant reduction in luciferase activity as the E/T ratio was increased compared with that of vehicle treatment (Fig. 8h). Cotreatment led to a further reduction in the luciferase activity compared with that of single treatments. Collectively, these results suggest that combination therapy with A3373 and αPD-L1 may enhance tumor regression via immune activation in the tumor environment.

## Discussion

PLD is convincingly linked to numerous diseases, including cancer^36^. Although PLD inhibition has been demonstrated to suppress cancer progression, the role and mechanism of PLD via immune modulation remain unknown. In the present study, we demonstrated that the pharmacological inhibition of PLD1 could modulate the immune microenvironment within tumors by inducing ICD, thereby promoting T-cell-based antitumor immune priming. We developed a new PLD inhibitor using CADD and pharmacophore studies that showed selectivity for PLD1. This may be due to the different binding modes between PLD1 and PLD2 in the docking study. The PLD inhibitor attenuated colitis-associated CRC and orthotopically injected tumors, probably by controlling multiple pathways, including Wnt signaling, phagocytosis checkpoints, and immune signaling. Furthermore, the inhibitor promoted the apoptosis of CRC cells but not normal colonic mucosa cells, was safe and exhibited no cardiotoxicity. Compound A3373 had a long half-life (3.2 h and 3.8 h after intravenous and oral dosing, respectively), high exposure (AUC_inf_ values; 6316.8 ng/h/mL and 13,647.5 ng/h/mL after intravenous and oral dosing, respectively) and potent oral bioavailability (108.0%) in an in vivo pharmacokinetic study. Thus, compound A3373 could be a highly potent and orally available anticancer chemotherapeutic agent. Moreover, we could not find any mortalities, clinical signs, changes in body weight, or necropsy findings when A3373 was orally administered to male and female mice at dose levels of 0, 500, 1000, and 2000 mg/kg of body weight, indicating that the approximate lethal dose in mice after a single oral dose of A3373 is over 2000 mg/kg in both female and male mice. Therefore, it seems that A3373 exhibits safety profiles in vivo.

Hyperactivation of Wnt/β-catenin is strongly associated with an immunosuppressive tumor microenvironment and immune checkpoint blockade resistance across cancer types^37^. PLD1 inhibition downregulated the Wnt/β-catenin signaling pathway. A therapeutic approach that can create an immunogenic environment within tumors involves the use of agents that induce ICD within the tumor. Accumulating evidence clearly underscores the ability of releasing DAMPs from dying cells to activate specific antitumor immune responses. We found that the PLD1 inhibitor elicited DAMP expression in tumor cells, which enhanced phagocytic activity and the subsequent processing and presentation of tumor antigens by macrophages. The accumulation of apoptotic cells, combined with the expression of DAMPs within tumors, is important for myeloid cell infiltration, activation, tumor antigen presentation, and T-cell priming. Our results are consistent with this scenario, as PLD1 inhibitor-induced apoptosis led to increased macrophage and CD8^+^ T-cell activation. Although the roles of PLD1 and PLD2 in cancer cells have been well studied, their functions in the tumor microenvironment have not yet been clarified. Recently, it has been reported that PLD2 in cytotoxic CD8^+^ T cells plays an important role in antitumor immunity^38^. Impaired CD8^+^ T-cell infiltration into the tumor microenvironment in *Pld2*^−/−^ mice promotes tumor growth. However, the possibility that PLD2 in other immune cells are also involved in suppression of tumor growth cannot be excluded. Thus, it is likely that PLD1 and PLD2 have differential roles in tumor immunity.

Wnt/β-catenin signaling impairs a multitude of immune functions in the tumor microenvironment by reducing the activation of cytotoxic T cells and upregulating the T-cell inhibitory PD-L1 as well as the “do not eat-me” signals^39^. PLD1 inhibition reduced the expression of the “do not eat-me” signals and upregulated the levels of “eat-me” signals, subsequently enhancing the phagocytosis of cancer cells by macrophages. We focused our efforts on identifying subsequent immunological changes and delineating the overall antitumor responses. PLD1 inhibition may exert anticancer activity via upregulation of cytotoxic CD8^+^ T cells and Th17 cells and downregulation of the Treg cell-mediated immune response. In fact, clinical responses to checkpoint inhibitors, such as αPD1/αPD-L1, are generally observed in cancers with increased tumor mutational burden, preexisting immunity, and higher expression of PD-L1. Combining checkpoint blockade with standard-of-care chemotherapy may provide the benefits of immunotherapy in earlier timelines, in which the cancer-immune set point might have a lower threshold for clinical response^40^. PD-L1 expression is associated with poor prognosis for patient survival in several tumor types^41^. High levels of PD-L1 are known to be related to resistance to antitumor therapies and are involved in the process of immune escape^42^. The combination of a PLD1 inhibitor with PD-L1 blockade treatment further promoted antitumor immunity via cytotoxic CD8^+^ T-cell activation in the tumor environment relative to monotherapy. An improved mechanistic understanding of drug-induced cell death and immunologic consequences may be the key to devising more efficacious and precise strategies to prevent and treat colon cancer. Drug-triggered ICD represents a promising approach to restore immunogenicity in tumors by engaging both innate and adaptive components to prime the tumor microenvironment to achieve a better antitumor response. Mechanism-guided combinations of ICD-inducing agents with immunotherapy and validated biomarkers will likely offer more tailored and durable benefits to patients with colon cancer. Collectively, our findings suggest that PLD1 inhibition may function as a potential immunomodulator and offer a promising new treatment modality for cancer immunotherapy.

## Supplementary information

Supplementary information
