# Supplementary material for: Inhibition of phospholipase D1 induces immunogenic cell death and potentiates cancer immunotherapy in colorectal cancer
Source: Exp Mol Med. 2022 Sep 21;54(9):1563–76. doi: 10.1038/s12276-022-00853-6 (PMC9535023; doi:10.1038/s12276-022-00853-6)
Supplement: Supplementary file 1 — Supplementary information [file 12276_2022_853_MOESM1_ESM.pdf]

Supplementary Information

Supplementary figures

Supplementary Fig. 1 Binding mode of A3373 with PLD2

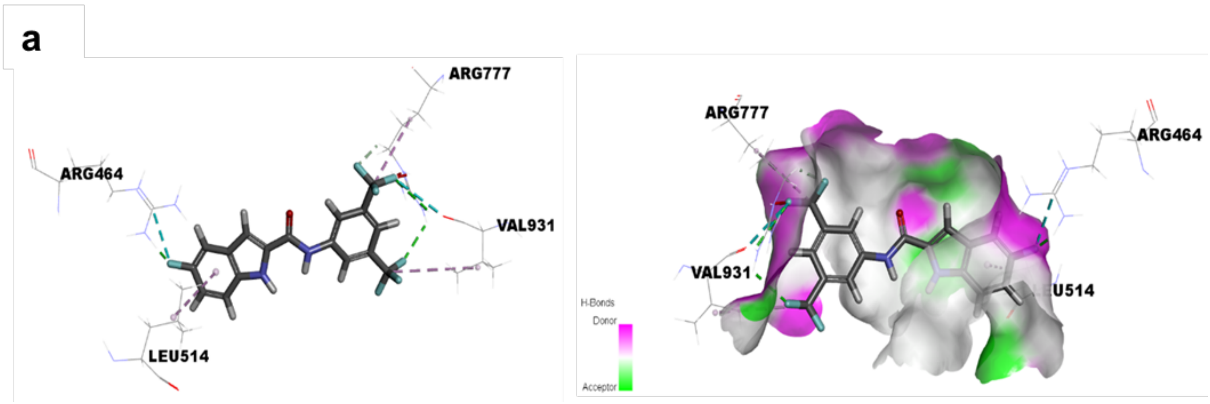

a Figure showing the binding site of PLD2 (PDB ID: 6OHS) on A3373. Structural simulation of the A3373-PLD2 complex showed that some residues were involved in binding with A3373, including non-bonded interactions (Leu514, Gln642, Phe643, and Val931) and hydrogen bonds (Arg464, Arg777, Val931, and Trp932). (green: hydrogen bond, pale pink: π-alkyl interaction, and carbon-halogen: cyan)

Supplementary Fig. 2 Effect of A3373 on the PLD activity in CRC cells

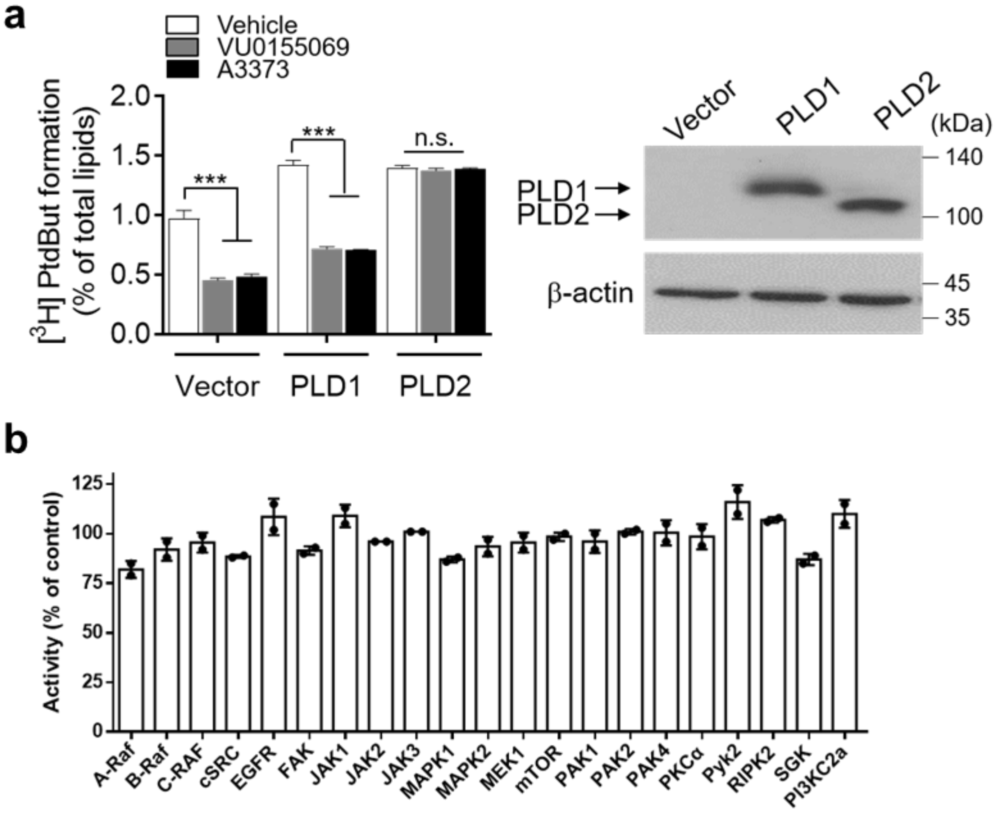

a HCT116 cells overexpressing PLD isozymes were treated with 2 μM of the indicated PLD inhibitors and PLD activity was determined. The expression of PLD isozymes was analyzed by immunoblotting. **b** The effect of A3373 on the activity of various kinases. The results are representative of at least three independent experiments and are presented as mean ± SD. ***, *P* < 0.001, n.s, not significant.

Supplementary Fig. 3 Effect of PLD1 inhibitor and 5-FU on the cell cycle and apoptosis in HCT116 cells

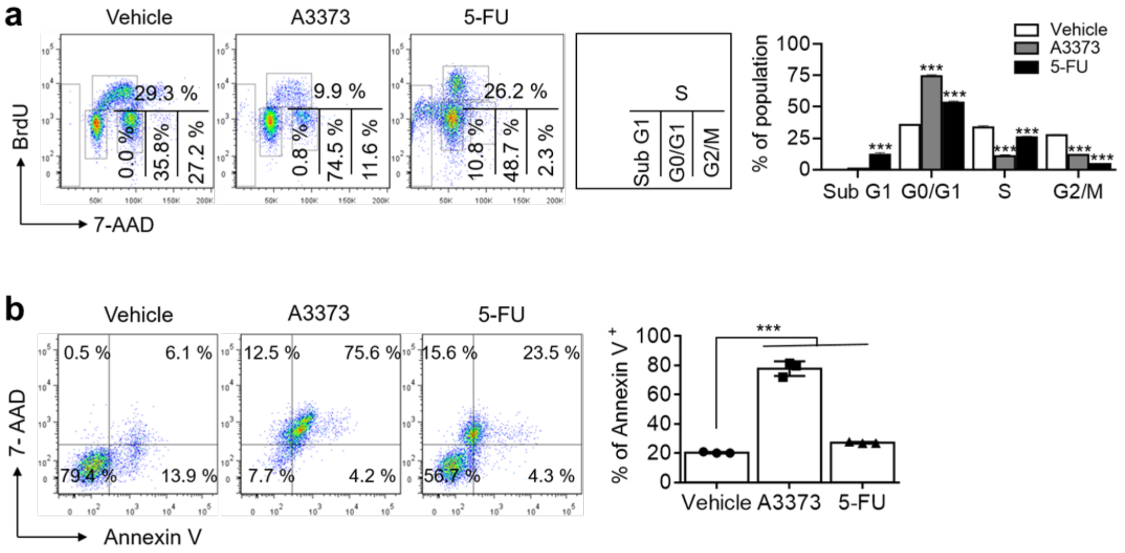

a HCT116 cells were treated with A3373 (2 μM) and 5-FU (10 μM) and cell cycle was analyzed. The population in the indicated cell cycle phases was quantified. **b** The indicated drugs were treated in HCT116 cells for 48 h, and apoptosis was analyzed in the presence of 1% FBS using annexin V and flow cytometry. The results are representative of at least three independent experiments and are presented as mean ± SD. ***, $P < 0.001$.

Supplementary Fig. 4 Effect of A3373 on Wnt/β-catenin signaling

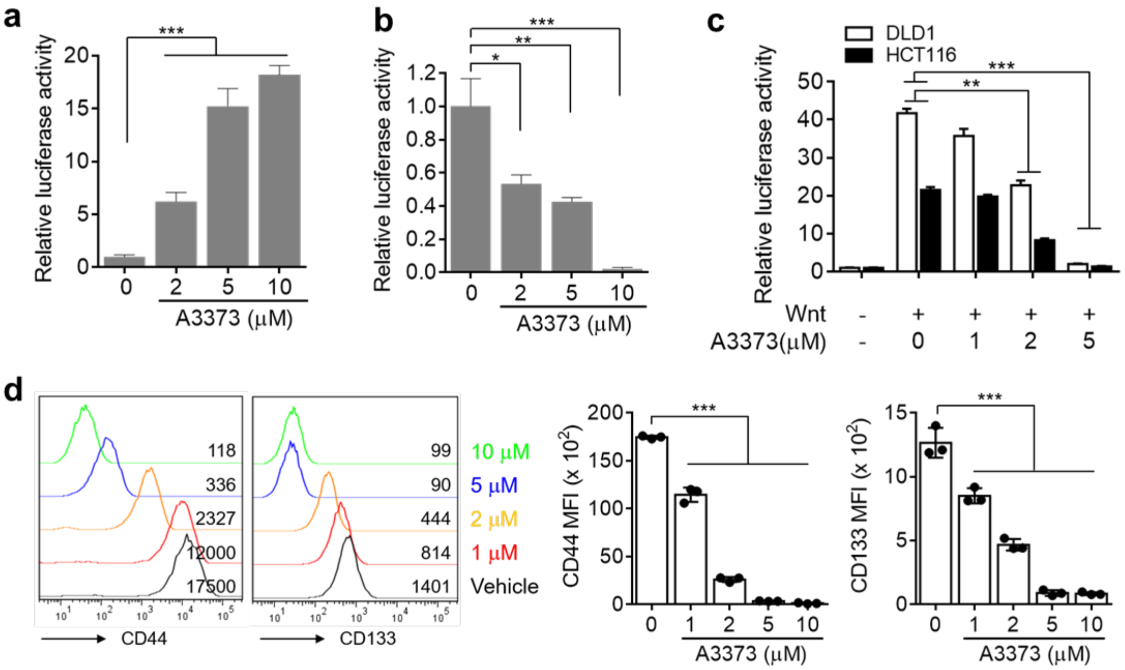

a Effect of A3373 on the activity of the miR-4496 promoter in HCT116 cells. **b** Effect of A3373 on the luciferase activity of the β-catenin 3′-UTR reporter. **c** Effect of A3373 on TCF transactivation in CRC cells with active status of the Wnt pathway. **d** Effect of A3373 on the expression of CD44 and CD136 in DLD1 cells, as analyzed by flow cytometry. The results are representative of at least three independent experiments and are presented as mean ± SD. *, $P < 0.05$; **, $P < 0.01$; ***, $P < 0.001$.

Supplementary Fig. 5 PLD1 regulates the expression of ‘don’t eat-me signals’ and ‘eat-me’ signal

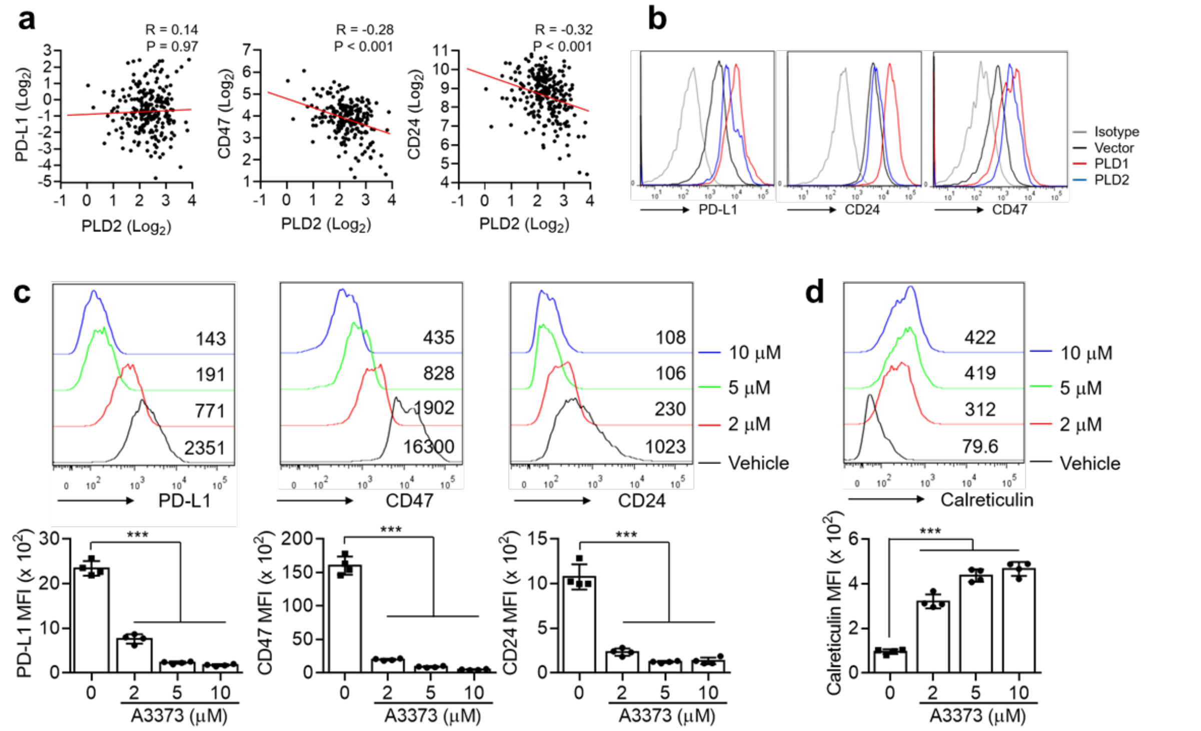

a Relationship of PLD2 expression with the levels of the ‘don’t eat-me’ signals’ in CRC as analyzed by TCGA data base. **b** Effect of ectopic expression of PLD isozymes on the levels of the ‘don’t eat-me’ signals. **c** and **d** Effect of A3373 on the levels of the ‘don’t eat-me' signals (**c**) and CRT (**d**) in DLD1 cells as analyzed by flow cytometry. The results are representative of at least three independent experiments and are presented as mean ± SD. ***, $P < 0.001$.

Supplementary Fig. 6 Effect of A3373 on the expression of proteins involved in Wnt/β-catenin and T cell-mediated signaling in colitis-associated CRC

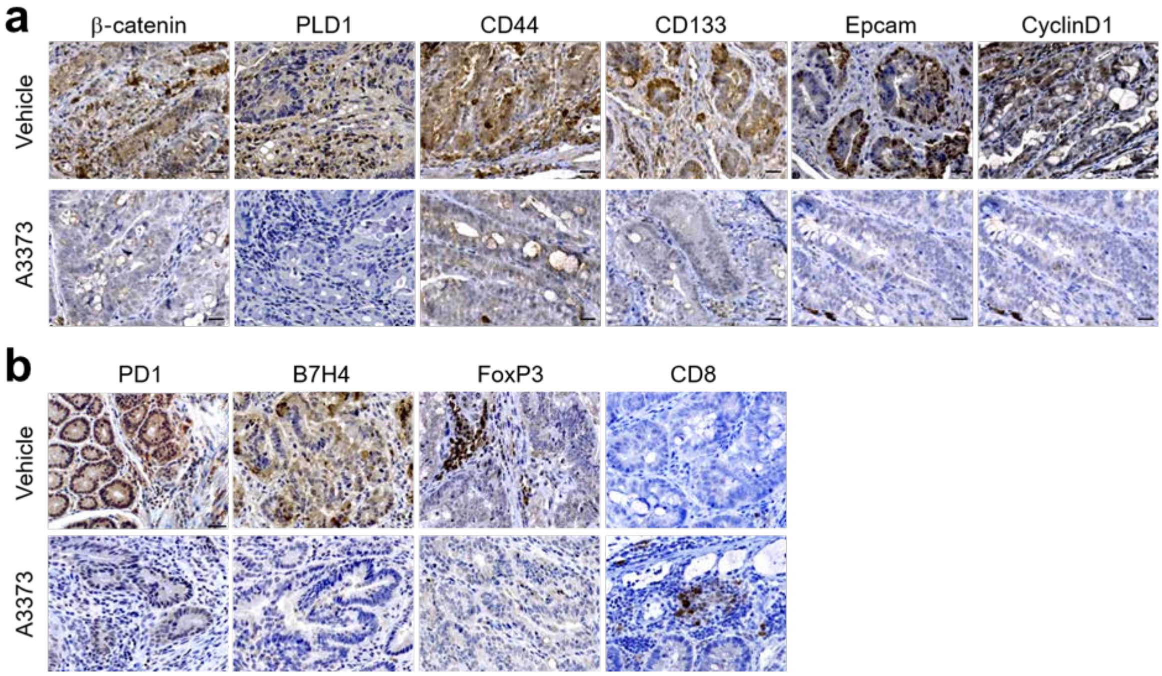

a IHC of β-catenin and its target proteins in tumor tissues from vehicle- or A3373-treated AOM/DSS mice. Scale Bar, 20 μm. **b** IHC of the indicated immune checkpoints and T cell markers in tumor tissues from vehicle- or A3373-treated AOM/DSS mice. Representative images were obtained from at least six fields. Scale Bar, 20 μm.

Supplementary Fig. 7 Effect of A3373 on the expression of ‘don’t eat-me’ signals, immune checkpoints, and T cell activation in colon cancer orthotropic model

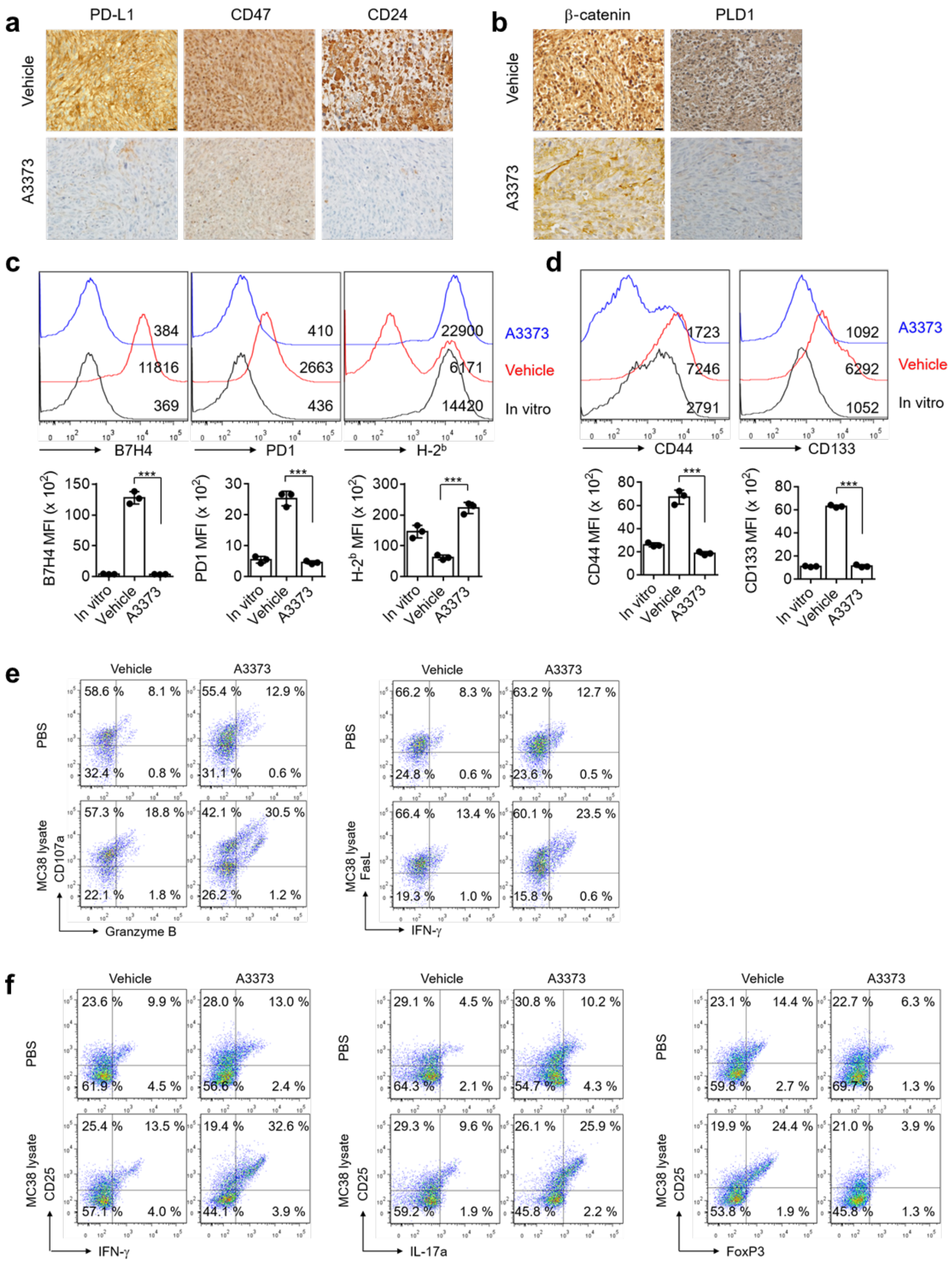

a IHC of ‘don’t eat-me’ signals. Scale Bar, 20 μm. **b** IHC for β-catenin and PLD1 in tumor tissues. Scale Bar, 20 μm. **c** and **d** The expression of the indicated immune checkpoints (**c**) and CIC markers (**d**) was analyzed by flow cytometry. **e** The populations of CD107a^{+} granzyme B^{+} and FasL^{+}IFN-γ^{+} in activated CD8^{+} T cells were analyzed by flow cytometry. **f** The population of IFN-γ^{+}, IL17a^{+} and Foxp3^{+} cells in activated CD4^{+} T cells was analyzed by flow cytometry. The results are representative of at least three independent experiments and are presented as mean ± SD. ***, $P < 0.001$. n.s, not significant.

Supplementary Fig. 8 Effect of A3373 and/or αPD-L1 on the expression of Foxp3, β-catenin, Ki67, and active caspase 3, and the phenotyping of macrophages in the colon cancer syngeneic model.

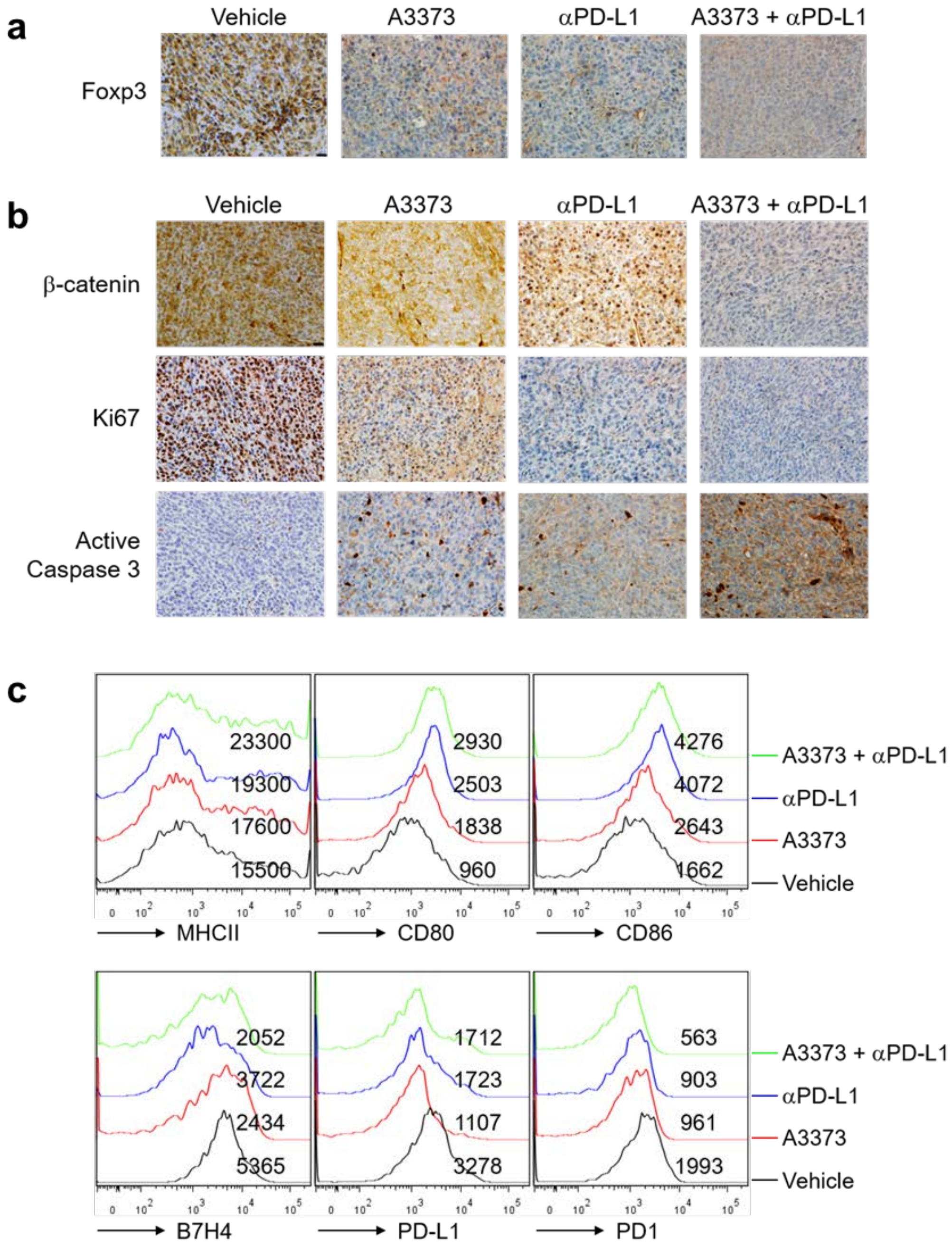

a and **b** IHC of Foxp3 (**a**), β-catenin, Ki67, and active caspase 3 (**b**) in the indicated drug(s)-treated tumor tissues. Scale Bar, 20 μm. **c,** Phenotypic analysis of macrophages infiltrating the tumors from drug-treated mice. Results are representative of at least three independent experiments.

Supplementary tables

Supplementary Table 1 Structure and evaluation of Fitvalue of various compounds

Compound	Structure	FitValue	Compound	Structure	FitValue
**B633**	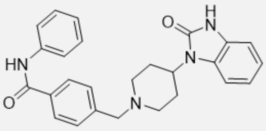	**2.94337**	**B681**	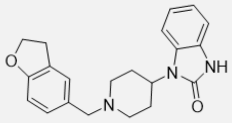	**2.96232**
**B635**	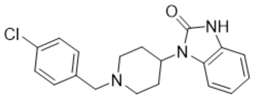	**3.66901**	**B711**	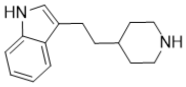	**2.84299**
**B636**	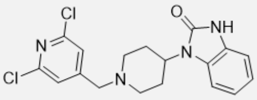	**2.98207**	**B724**	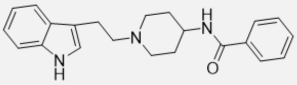	**3.51029**
**B639**	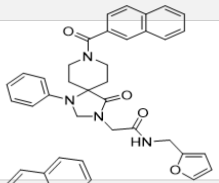	**2.68914**	**B728**	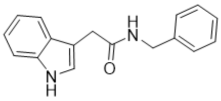	**2.97704**
**B642**	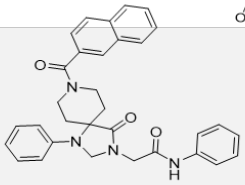	**2.67822**	**B730**	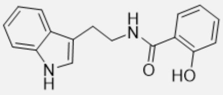	**2.64816**

The fitness values of the compounds were evaluated in a virtual screening process based on the pharmacophores of VU0155069 in complex with PLD1, and the structure of the compounds is shown.

Supplementary Table 2 Structure of synthetic compounds derived from B728

Compound	Structure	Compound	Structure
A2666	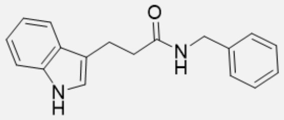	A3381	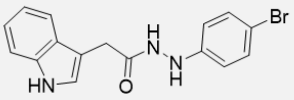
A3271	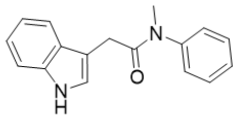	A3491	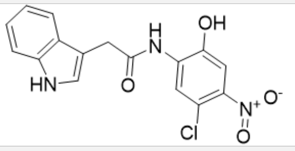
A3272	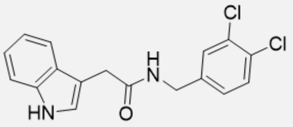	A3667	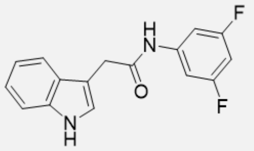
A3324	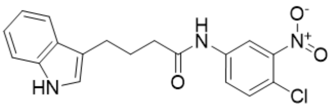	A3669	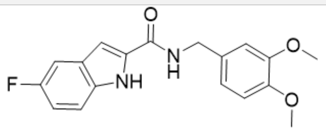
A3325	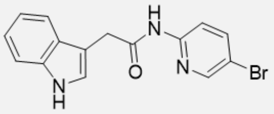	A4145	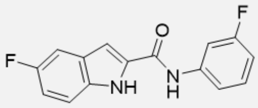
A3373	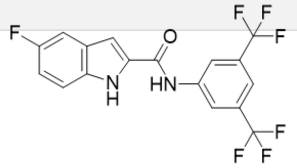	VU0155069	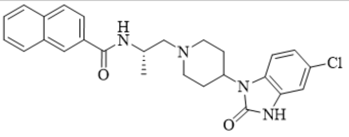

Supplementary Table 3 Binding energies of PLD inhibitors

compound	Binding energy (kcal/mol)	
	PLD1 (6OHR)	PLD2 (6OHS)
A3373	-193.46	-151.13
VU0155069	-181.18	-192.83

The binding model of A3373 and VU0155069 with PLD1 or PLD2 was performed in a docking study, and the binding energies were calculated using the Dock Ligands and Calculate Binding Energies protocols.

Supplementary Table 4. Plasma pharmacokinetics of A3373

Parameter	Unit	IV			PO		
		mean	SD	N	mean	SD	N
Dose	mg/kg	5		3	10		3
$t_{1/2}$	h	3.2	0.4	3	3.8	0.2	3
t_{max}	h	NA			0.5	0.0	3
C_{max}	ng/mL	NA			2629.6	182.6	3
AUC_{last}	ng.h/mL	6299.6	362.1	3	13485.4	1455.0	3
AUC_{inf}	ng.h/mL	6316.8	363.7	3	13647.5	1506.9	3
MRT	h	2.6	0.1	3	5.6	0.4	3
V_z	L/kg	3.7	0.6	3	NA		
CL	L/h/kg	0.8	0.1	3	NA		
V_{ss}	L/kg	2.1	0.1	3	NA		
F	%	NA			108.0	11.9	3

The pharmacokinetic parameters were estimated with non-compartmental analysis using PKSolver, based on the concentration-time profiles of A3373 in the plasma samples following single IV (5 mg/kg) and PO (10 mg/kg) dose in mice (n=3). Abbreviations: $T_{1/2}$, elimination half life; T_{max}, time to peak concentration; AUC, area under the concentration–time curve; MRT, mean residence time; V_z, the volume in the terminal state; CL, clearance; V_{ss}, steady-state volume of distribution; F, absolute bioavailability.

Supplementary Table 5

Antibody	Source
PLD1	Santa Cruz
β-catenin	Santa Cruz
CD133	MYBioSource
CD44	Santa Cruz
c-Myc	abcam
Cyclin D1	Santa Cruz
TCF4	Santa Cruz
CD24	Santa Cruz
CD47	abcam
PD-L1	abcam
β-actin	Santa Cruz
Active caspase 3	Cell Signaling
Ki67	abcam
CD31	abcam
Calreticulin	abcam
Epcam	Santa Cruz
PD1	Santa Cruz
B7H4	abcam
FoxP3	Santa Cruz
**FACS antibody**	**Source**
phycoerythrin (PE)-conjugated anti-human CD133	Miltenyi Biotec
allophycocyanin (APC)-conjugated anti-human CD44	BD Biosciences
APC-conjugated anti-human CD47	Thermo Fisher Scientific
APC-conjugated anti-human CD24	Thermo Fisher Scientific
PE-conjugated anti-human PD-L1	Thermo Fisher Scientific
PE-conjugated anti-human B7H4	Thermo Fisher Scientific
PE-conjugated anti-human PD1	Thermo Fisher Scientific
PE-conjugated anti-human calreticulin	Thermo Fisher Scientific
PE-conjugated anti-mouse CD133	Thermo Fisher Scientific

APC-conjugated anti-mouse CD44	Thermo Fisher Scientific
APC-conjugated anti-mouse CD47	Thermo Fisher Scientific
APC-conjugated anti-mouse CD24	Thermo Fisher Scientific
PE-conjugated anti-mouse PD-L1	Thermo Fisher Scientific
PE-conjugated anti-mouse B7H4	Thermo Fisher Scientific
PE-conjugated anti-mouse PD1	Thermo Fisher Scientific
APC-conjugated anti-mouse CTLA4	Invitrogen
APC Cy7-conjugated anti-mouse CD107a	BioLegend
APC-conjugated anti-mouse granzyme B	Invitrogen
APC Cy7-conjugated anti-mouse FasL	Invitrogen
APC-conjugated anti-mouse IFN-γ	BD Biosciences
PerCP-Cy5.5-conjugated anti-mouse CD4	BD Biosciences
PerCP-Cy5.5-conjugated anti-mouse CD8	BD Biosciences
FITC-conjugated anti-mouse CD25	BD Biosciences
PE-Cy7-conjugated anti-mouse TCRβ	BD Biosciences
APC Cy7-conjugated anti-mouse IL-17a	BioLegend
PE-conjugated anti-mouse FoxP3	Invitrogen
PE-conjugated anti-mouse MHCII	BioLegend
PE-conjugated anti-mouse CD80	BioLegend
PE-conjugated anti-mouse CD86	BioLegend
PeCy7-conjugated anti-mouse F4/80	BioLegend
PerCP-Cy5.5-conjugated anti-mouse CD11b	BD Biosciences
FITC-conjugated anti-mouse CD45	Invitrogen

Supplementary materials and methods

Docking study and pharmacophore modeling

All molecular docking analyses were performed using Discovery Studio 2020 software (Biovia) adopting the CHARMm force field. The X-ray crystal structures of hPLD1 and hPLD2 (Protein Data Bank code 6OHR and 6OHS) were obtained from the RCSB protein databank and refined by removing water molecules and adding hydrogen atoms to the entire enzyme. Ligand-fit docking was performed to dock ligands. Crystallographic ligands (PDB ID: MKG and MJY) were used to determine the binding sites of 6OHR and 6OHS, respectively. VU0155069 docked with PLD1, and the results of the docking study were used to generate pharmacophore models using the Auto Pharmacophore Generation protocol. Compounds from the in-house library (3,200) were passed directly for quick pharmacophore features using the screening library protocol. The fitted hit compounds were arranged based on the pharmacophore fit score (FitValue). A3373 and VU0155069 were docked to the binding sites of proteins (PLD1 and PLD2) by Dock Ligands the docking method, and 30 poses were generated for each. Based on the docking results, the binding energy of the most predictive binding mode was calculated using the calculated binding energy protocol.

Kinase panel assay

In vitro kinase activity assays of A3373 were performed using the 21 kinase KinaseProfilerTM (eurofins). Kinase activity was determined after incubation with 1 μM A3373, according to the KinaseProfilerTM Service standard assay protocols.

Animal experiments

The AOM/DSS colorectal cancer model was established using 6-weeks-old C57BL/6 male mice (Koatech). Mice were given a single intraperitoneal (IP) injection of the mutagen AOM (10 mg/kg), after which they were given three periods drinking water containing 2.5% DSS for 5 days, followed by 15 days of normal water. AOM/DSS-induced mice were given either vehicle (PBS containing 8% DMSO and 1% Tween 80) or 10 mg/kg A3373 compound every day by oral administration for 45 days.

A colon cancer orthotropic model was established using 6-weeks-old C57BL/6 male mice. Mice were administered a single IP injection of 2,2,2-Tribromoethanol (Avertin, Sigma-aldrich), an anesthetic. The abdomen was shaved and the surgical site was draped in a sterile

manner. The incision was extended to 2–3 cm, and the cecum was exposed. 2 × 10^5 MC38-GFP-Luciferase cells were suspended in 50 µL PBS and injected into the cecum. Seven days after cell injection, 10 mg/kg A3373 was orally administered to the mice daily for 4 weeks.

A colon cancer syngeneic model was established using 6-weeks-old C57BL/6 male mice. 2 × 10^5 MC38 cells were suspended in 50 µl PBS and subcutaneously injected into the flanks of the mice. After 7 days of cell injection, mice were randomized into four groups and all groups were treated as follows for 2 weeks: vehicle group, mice were IP administered vehicle every day; A3373 group, mice were IP administrated with 10 mg/kg A3373 every day; αPD-L1 antibody group, mice were IP administrated with 5 mg/kg anti-mouse PD-L1 (BioXcell) every two days; A3373 + αPD-L1 antibody group, mice received A3373 (10 mg/kg) daily with anti-mouse PD-L1 (5 mg/kg) every 2 days. Tumor volume was measured in MC38 cells subcutaneous injected mice every 2 days after 7 days of cell injection. External tumor measurements were made using precision calipers, and tumor volume (TV) was calculated according to the following formula: $TV = (W \times L^2)/2$ (W = width and L = length).

All mice were housed at 25°C under a 12:12 hours light/dark cycle. All animal procedures were performed in accordance with the institutional guidelines for animal research and were approved by the Institutional Animal Care and Use Committee of Yonsei University (IACUC-202008-1116-01).

Flow cytometry

In a colon cancer orthotropic model, tumor-infiltrating lymphocytes (TILs) were isolated from the tumor tissue 30 days after cell injection. Tumors were enzymatically dissociated in RPMI media containing 10% FBS, 200 mg/mL collagenase IV (Sigma-Aldrich), 20 mg/mL hyaluronidase (Sigma-Aldrich), and 20 mg/mL DNase I (Sigma Aldrich) at 37 °C with stirring. After 20 minutes of incubation, 0.1 M EDTA was added to prevent clumping. Cells were filtered through a 40 µm nylon mesh, single-cell suspensions were counted and used for the experiments. Finally, the TILs were resuspended in PBS containing 1% FBS. For the distribution of immune cells, anti-mouse CD45 microbeads were used according to the manufacturer's instructions (Miltenyi Biotech). Isolated tumor cells were cultured for one passage to change the immunocompromised phenotype and assessed using flow cytometry. Splenocytes were isolated from mouse spleens, seeded at 5 × 10^6 cells, and stimulated with 10 µg/mL MC38 GFP-Luciferase cell lysates. For intracellular cytokine staining, cells were

additionally treated with brefeldin A (BD Pharmingen) 16 hours prior to analysis to block the intracellular transport of cytokines from the endoplasmic reticulum to the Golgi complex. CD8 T cells were subsequently stained with PerCP-Cy5.5-conjugated anti-mouse CD8, PE-Cy7-conjugated anti-mouse TCRβ, FITC-conjugated anti-mouse CD25, APC Cy7-conjugated anti-mouse FasL, and APC Cy7-conjugated anti-mouse CD107a, fixed and permeabilized with a Cytofix/Cytoperm Kit (BD Pharmingen) following the manufacturer's instructions. Cells were incubated with APC-conjugated anti-mouse IFNγ-APC-conjugated anti-mouse granzyme B. For CD4 T cell staining, cells were stained with PerCP-Cy5.5-conjugated anti-mouse CD4, PE-Cy7-conjugated anti-mouse TCR,β and FITC-conjugated anti-mouse CD25, fixed and permeabilized. After permeabilization, the cells were labeled with APC-conjugated anti-mouse FoxP3, APC Cy7-conjugated anti-mouse IL-17a, and APC-conjugated anti-mouse IFN-γ antibody. The cells were analyzed using a FACSAria III (BD Biosciences). Data analysis was performed using FlowJo v10.0 (BD Biosciences).

Immunohistochemistry and histology of tumor

At the end of the animal experiments, the mice were sacrificed, and tumor tissues were harvested. Tissues were fixed with 4% paraformaldehyde, embedded in paraffin, sectioned, and stained with Hematoxylin and eosin (H&E). IHC staining was done using primary antibodies as shown in Supplementary Table S4. All histopathological images were taken with Olympus BX53 microscope and cellSens image acquisition software (Olympus)

MC38-GFP-Luciferase specific CD8 T cell cytotoxicity assay

Mice were subjected to experiments at 8 weeks of age and immunized by subcutaneous injection at the base of the tail. The MC38-GFP-Luciferase cell lysates were prepared to provide a total of 100 μg/dose. Complete Freund's adjuvant (CFA, Sigma-Aldrich) was used at 100 μL/dose to make the emulsion, keeping their ratios at 1:1, and delivered in a total volume of 0.1 mL. After one week of immunization, the mice were subjected to secondary immunization. The same dose of lysates was emulated with CFA with the same dose of incomplete Freund's adjuvant (IFA, Sigma Aldrich), immunized by subcutaneous injection at the base of the tail. After 1 week of secondary immunization, all lymphoid organs were harvested to isolate immune cells. Isolated immune cells (5×10^6) were stimulated with 10 μg/mL of MC38-GFP-Luciferase cell lysate for two days. Restimulated immune cells were

isolated from CD8+ T cells using anti-mouse CD8 microbeads according to the manufacturer's protocol (Miltenyi Biotech). An *in vitro* cytotoxicity assay was performed according to the manufacturer's instructions[1]. MC38 GFP-Luciferase cells were seeded in 96-well round-bottomed plates (5×10^3 cells/well). After overnight incubation, the medium was replaced with 100 μL of fresh medium containing 2 or 5 μM of vehicle or A3373 . The luciferase-expressing tumor cells were incubated in A3373-containing medium at 37 °C, 5% CO_2 for 24 hours . Effector CD8+ T cells were added at a density of 5×10^4 cells/well in vehicle or A3373 treated tumor cells for 6 hours. After incubation, D-luciferin (potassium salt, PerkinElmer) was added to each well at 150 μg/mL in medium for 10 minutes before calculating with the GloMax (Promega).

In vivo pharmacokinetics

Male ICR mice (8 weeks old, 30~35 g) were acclimated to the testing facility in a temperature and humidity controlled condition for approximately a week prior to the study. 1 mg/mL of A3373 solution in dimethylacetamide/tween 80/saline (10/10/80 vol%) was dosed orally (10 mg/kg in a volume of 10 mL/kg) or by tail vein injection (5 mg/kg in a volume of 5 mL/kg) to the animals. About 40 μL of blood samples were collected in BD Microtainer plasma separator tubes at selected times through the saphenous vein over 24 hours post-dosing. Blood samples were centrifuged at 6,000 x g for 5 minutes to separate plasma and stored in a freezer until analyzed. Protein precipitation was conducted on 15 μL aliquot of plasma samples with 3 volumes of acetonitrile containing glipizide as analytical internal standard. After centrifugation, the supernatant was analyzed by Agilent 6460 QQQ LC-MS/MS system in a negative MRM mode. Briefly, chromatography was performed on a Phenomenex Kinetex C_{18} column (2.1 × 50 mm, 2.6 μm) with a linear gradient of the mobile phases 10 mM aqueous ammonium acetate and acetonitrile. Detection of the analyte ions was performed in negative multiple reaction monitoring mode, monitoring the precursor>product ion transitions of m/z 389>134 and 444>319 for A3373 and the IS, respectively.

Chemistry

Chemicals obtained from commercial suppliers were used without further purification. All reactions were monitored by thin-layer chromatography on pre-coated silica gel 60 F254 (mesh) (E. Merck, Mumbai, India), and spots were visualized under UV light (254 nm). Flash column chromatography was performed using silica (Merck EM9385; 230–400 mesh). ^1H and ^{13}C

nuclear magnetic resonance (NMR) (Agilent Technologies) spectra were recorded at 400 and 100 MHz, respectively. Proton and carbon chemical shifts are expressed in ppm relative to the internal tetramethylsilane. The coupling constants (*J*) are expressed in Hertz. Splitting patterns are presented as s, singlet; d, doublet; t, triplet; q, quartet; dd, double doublets; m, multiplet; br, broad. Liquid chromatography-mass spectrometry (LC-MS) spectra were obtained with an electrospray ionization (ESI) probe using a Shimadzu LCMS-2020 instrument with an Agilent C18 column (50*4.6 mm, 5 μm). The detected ion peaks were m/z in the positive and negative scan modes, where m represents the molecular weight of the compound and z represents the charge (number of protons). To determine the purity of the final compounds, HPLC experiments were conducted using an Agilent analytical column eclipse-XDB-C_{18} (150*4.6 mm, 5 μm) on a Shimadzu HPLC-2010 instrument.

General procedure

A mixture of the corresponding indole-2-carboxylic acid (1 equiv), corresponding amine (1 equiv), EDC·HCl (2 equiv), HOBt·H_2O (2 equiv), and DIPEA (3 equiv) in CH_2Cl_2 (0.1 M) was stirred overnight at 20 °C. The reaction mixture was concentrated under reduced pressure. The resulting residue was purified by flash column chromatography (ethyl acetate: hexane = 1: 5 – 1: 1) to obtain the compound.

N-benzyl-3-(1H-indol-3-yl)propanamide (A2666)

It was obtained as white solid (72.5% yield). ^{1}H NMR (400 MHz, DMSO-*d6*) δ 10.76 (s, 1H), 8.32 (t, 1H, *J* = 5.4 Hz), 7.53 (d, 1H, *J* = 7.6 Hz), 7.33 (d, 1H, *J* = 8.0 Hz), 7.25 (t, 2H, *J* = 7.2 Hz), 7.19 (d, 1H, *J* = 7.2 Hz), 7.14 (d, 2H, *J* = 7.2 Hz), 7.08 (s, 1H), 7.05 (t, 1H, *J* = 7.6 Hz), 6.95 (t, 1H, *J* = 7.2 Hz), 4.26 (d, 2H, *J* = 5.6 Hz), 2.96 (t, 2H, *J* = 7.4 Hz), 2.51 (t, 2H, *J* = 7.4 Hz). ^{13}C NMR (100 MHz, DMSO-*d6*) δ 172.3, 140.0, 136.7, 128.6, 128.6, 127.6, 127.6, 127.5, 127.0, 122.7, 121.3, 118.8, 118.6, 114.2, 111.7, 42.4, 36.8, 21.5. RP-HPLC (254 nm): 99%. LC/MS (ESI, *m/z*) 279 (MH^+) and 301 (MNa^+).

2-(1H-indol-3-yl)-N-methyl-N-phenylacetamide (A3271)

It was obtained as yellow solid (74.2% yield). ^{1}H NMR (400 MHz, DMSO-*d6*) δ 10.80 (s, 1H), 7.48 – 7.37 (m, 2H), 7.37 – 7.19 (m, 5H), 7.01 (t, 1H, *J* = 7.5 Hz), 6.90 (t, 2H, *J* = 6.8 Hz), 3.46 (s, 2H), 3.16 (s, 3H). RP-HPLC (254 nm], 95%). LC-MS (ESI, *m/z*) 265 (MH^+) and 287 (MNa^+).

N-(3,4-dichlorobenzyl)-2-(1H-indol-3-yl)acetamide (A3272)

It was obtained as yellow solid (51.9% yield). ^{1}H NMR (400 MHz, DMSO-*d6*) δ 10.87 (s, 1H), 8.43 (t, 1H, *J* = 5.9 Hz), 7.53 – 7.47 (m, 2H), 7.40 (d, 1H, *J* = 0.9 Hz), 7.32 (d, 1H, *J* = 8.1 Hz), 7.21 – 7.15 (m, 2H), 7.04 (t, 1H, *J* = 7.5 Hz), 6.94 (t, 1H, *J* = 7.4 Hz), 4.23 (d, 2H, *J* = 6.0 Hz), 3.56 (s, 2H). ^{13}C NMR (100 MHz, DMSO-*d6*) δ 171.4, 141.4, 136.5, 131.2, 130.7, 129.5, 129.4, 127.9, 127.5, 124.3, 121.4, 119.0, 118.7, 111.7, 109.0, 41.5, 33.1. RP-HPLC (254 nm): 96%. LC/MS (ESI, *m/z*) 331 (MH^-).

N-(4-chloro-3-nitrophenyl)-4-(1H-indol-3-yl)butanamide (A3324)

It was obtained as deep-yellow solid (62.2% yield). ^{1}H NMR (400 MHz, DMSO-*d6*) δ 10.75 (s, 1H), 10.40 (s, 1H), 8.39 (d, 1H, *J* = 2.4 Hz), 7.73 (dd, 1H, *J* = 8.9, 2.4 Hz), 7.64 (d, 1H, *J* = 8.8 Hz), 7.49 (d, 1H, *J* = 7.8 Hz), 7.30 (d, 1H, *J* = 8.1 Hz), 7.10 (d, 1H, *J* = 1.5 Hz), 7.02 (t, 1H, *J* = 7.5 Hz), 6.93 (t, 1H, *J* = 7.4 Hz), 2.71 (t, 2H, *J* = 7.4 Hz), 2.38 (t, 2H, *J* = 7.4 Hz), 2.01 – 1.90 (m, 2H, *J* = 7.4 Hz). ^{13}C NMR (100 MHz, DMSO-*d6*) δ 172.5, 147.6, 139.5, 136.7, 132.3, 127.5, 124.2, 122.8, 121.2, 118.7, 118.5, 118.4, 115.6, 114.2, 111.7, 36.5, 25.9, 24.6. RP-HPLC (254 nm): 99%. LC/MS (ESI, *m/z*) 356 (MH^-), 380 (MNa^+).

N-(5-bromopyridin-2-yl)-2-(1H-indol-3-yl)acetamide (A3325)

It was obtained as gray solid (75.7% yield). ^{1}H NMR (400 MHz, DMSO-*d6*) δ 10.90 (s, 1H), 10.72 (s, 1H), 8.39 (d, 1H, *J* = 2.5 Hz), 8.03 (d, 1H, *J* = 8.9 Hz), 7.93 (dd, 1H, *J* = 8.9, 2.5 Hz), 7.57 (d, 1H, *J* = 7.9 Hz), 7.32 (d, 1H, *J* = 8.1 Hz), 7.25 (d, 1H, *J* = 1.7 Hz), 7.04 (t, 1H, *J* = 7.5 Hz), 6.95 (t, 1H, *J* = 7.4 Hz), 3.79 (s, 2H). ^{13}C NMR (100 MHz, DMSO-*d6*) δ 171.1, 151.5, 148.8, 140.9, 136.5, 127.5, 124.5, 121.4, 119.1, 118.8, 115.3, 113.6, 111.8, 108.4, 33.9. RP-HPLC (254 nm], 98%). LC/MS (ESI, *m/z*) 330 (MH^+), 332 (MH^+).

N-(3,5-bis(trifluoromethyl)phenyl)-5-fluoro-1H-indole-2-carboxamide (A3373)

It was obtained as white solid (68.7% yield). ^{1}H NMR (400 MHz, DMSO-*d6*) δ 11.93 (s, 1H), 10.79 (s, 1H), 8.50 (s, 2H), 7.78 (s, 1H), 7.51 – 7.41 (m, 3H), 7.09 (td, 1H, *J* = 9.3, 2.5 Hz). RP-HPLC (254 nm): 99%. LC/MS (ESI, *m/z*) 389 (MH^-).

N'-(4-bromophenyl)-2-(1H-indol-3-yl)acetohydrazide (A3381)

It was obtained as yellow solid (69.5% yield). ^{1}H NMR (400 MHz, DMSO-*d6*) δ 10.88 (s, 1H), 9.83 (s, 1H), 7.91 (s, 1H), 7.59 (d, 1H, *J* = 7.8 Hz), 7.33 (d, 1H, *J* = 8.0 Hz), 7.19 (d, 3H, *J* =

8.6 Hz), 7.06 (t, 1H, *J* = 7.4 Hz), 6.98 (t, 1H, *J* = 7.4 Hz), 6.60 (d, 2H, *J* = 8.3 Hz), 3.58 (s, 2H). RP-HPLC (254 nm], 97%). LC/MS (ESI, *m/z*) 342 (MH^-).

N-(5-chloro-2-hydroxy-4-nitrophenyl)-2-(1H-indol-3-yl)acetamide (A3491)

It was obtained as yellow solid (70.8% yield). RP-HPLC (254 nm): 99%. LC/MS (ESI, *m/z*) 346 (MH^+)

N-(3,5-difluorophenyl)-2-(1H-indol-3-yl)acetamide (A3667)

It was obtained as yellow solid (59.5% yield). ^{1}H NMR (400 MHz, DMSO-*d6*) δ 10.91 (s, 1H), 10.45 (s, 1H), 7.54 (d, 1H, *J* = 7.9 Hz), 7.34 – 7.28 (m, 3H), 7.23 (d, 1H, *J* = 1.8 Hz), 7.04 (t, 1H, *J* = 7.5 Hz), 6.95 (t, 1H, *J* = 7.4 Hz), 6.85 (t, 1H, *J* = 9.4 Hz), 3.71 (s, 2H). RP-HPLC (254 nm): 99%. LC/MS (ESI, *m/z*) 285 (MH^-).

N-(3,4-dimethoxybenzyl)-5-fluoro-1H-indole-2-carboxamide (A3669)

It was obtained as white solid (63.6% yield). ^{1}H NMR (400 MHz, DMSO-*d6*) δ 11.68 (s, 1H), 8.97 (t, 1H, *J* = 5.9 Hz), 7.41 – 7.34 (m, 2H), 7.13 (s, 1H), 7.00 (td, 1H, *J* = 9.3, 2.4 Hz), 6.93 (s, 1H), 6.89 – 6.80 (m, 2H), 4.40 (d, 2H, *J* = 5.9 Hz), 3.70 (s, 3H), 3.69 (s, 3H). RP-HPLC (254 nm], 98%). LC/MS (ESI, *m/z*) 327 (MH^-).

5-fluoro-N-(3-fluorophenyl)-1H-indole-2-carboxamide (A4145)

It was obtained as white solid (66.5% yield). ^{1}H NMR (400 MHz, DMSO-*d6*) δ 11.91 (s, 1H), 10.43 (s, 1H), 7.79 (dt, 1H, *J* = 11.8, 2.1 Hz), 7.59 (dd, 1H, *J* = 8.2, 0.9 Hz), 7.52 – 7.38 (m, 4H), 7.11 (td, 1H, *J* = 9.3, 2.5 Hz), 6.95 (td, 1H, *J* = 8.4, 2.0 Hz). RP-HPLC (254 nm): 99%. LC/MS (ESI, *m/z*) 273 (MH^+), 271 (MH^-).

N-(3,5-bis(trifluoromethyl)phenyl)-5-(2-(5-((3aS,4S,6aR)-2-oxohexahydro-1H-thieno[3,4-d]imidazol-4-yl)pentanoyl)hydrazine-1-carbonyl)-1H-indole-2-carboxamide (A4333)

It was obtained as white solid (25.0% yield). LC/MS (ESI, *m/z*) 657 (MH^+), 655 (MH^-).

References

1. Tseng, C. -W. et al. Pretreatment with Cisplatin Enhances E7-Specific CD8+ T-Cell–Mediated Antitumor Immunity Induced by DNA Vaccination. *Clin. Cancer Res.* **14,** 3185-3192 (2008).
